# Pre-innervated tissue-engineered muscle promotes a pro-regenerative microenvironment following volumetric muscle loss

**DOI:** 10.1038/s42003-020-1056-4

**Published:** 2020-06-25

**Authors:** Suradip Das, Kevin D. Browne, Franco A. Laimo, Joseph C. Maggiore, Melanie C. Hilman, Halimulati Kaisaier, Carlos A. Aguilar, Zarina S. Ali, Foteini Mourkioti, D. Kacy Cullen

**Affiliations:** 1 Center for Brain Injury & Repair, Department of Neurosurgery, Perelman School of Medicine, University of Pennsylvania, Philadelphia, PA USA; 2 Center for Neurotrauma, Neurodegeneration & Restoration, Corporal Michael J. Crescenz Veterans Affairs Medical Center, Philadelphia, PA USA; 3 Department of Bioengineering, School of Engineering and Applied Science, University of Pennsylvania, Philadelphia, PA USA; 4 Department of Biomedical Engineering, University of Michigan, Ann Arbor, MI USA; 5 Department of Orthopedic Surgery, Perelman School of Medicine, University of Pennsylvania, Philadelphia, PA USA; 6 Department of Cell and Developmental Biology, Perelman School of Medicine, University of Pennsylvania, Philadelphia, PA USA; 7 Penn Institute for Regenerative Medicine, Musculoskeletal Program, Perelman School of Medicine, University of Pennsylvania, Philadelphia, PA USA

**Keywords:** Tissue engineering, Implants

## Abstract

Volumetric muscle loss (VML) is the traumatic or surgical loss of skeletal muscle beyond the inherent regenerative capacity of the body, generally leading to severe functional deficit. Formation of appropriate somato-motor innervations remains one of the biggest challenges for both autologous grafts as well as tissue-engineered muscle constructs. We aim to address this challenge by developing pre-innervated tissue-engineered muscle comprised of long aligned networks of spinal motor neurons and skeletal myocytes on aligned nanofibrous scaffolds. Motor neurons led to enhanced differentiation and maturation of skeletal myocytes in vitro. These pre-innervated tissue-engineered muscle constructs when implanted in a rat VML model significantly increased satellite cell density, neuromuscular junction maintenance, graft revascularization, and muscle volume over three weeks as compared to myocyte-only constructs and nanofiber scaffolds alone. These pro-regenerative effects may enhance functional neuromuscular regeneration following VML, thereby improving the levels of functional recovery following these devastating injuries.

## Abstract (web summary)

Das et al. create a bioengineered construct combining nerve and muscle cells on a biomimetic scaffold to improve recovery from volumetric muscle loss (VML). When implanted into a rat VML model, they report an increase in in vitro parameters of muscle fiber development and formation of neuromuscular junctions, which support in vivo observations of improved implanted cell survival and vascularization.

## Introduction

Innervation plays a crucial role in the development, maturation, and functioning of different muscles in the body and yet remains largely unexplored in tissue engineering studies related to cardiac, smooth, or skeletal muscle. There are limited reports showing the importance of re-innervation in a transplanted heart or in a tissue-engineered urinary bladder in order to achieve complete functional recovery^1–3^. Although the concept of “pre-innervation” (akin to pre-vascularization), wherein appropriate neuronal populations are cultured along with relevant muscle cells has been established for a long time in vitro^4–7^, there are limited animal studies on the implications of pre-innervated constructs on muscle regeneration^8^. The present study is focused toward exploring the effect of pre-innervated implants on host muscle pathophysiology in a severe muscle trauma model like volumetric muscle loss (VML).

VML is defined as traumatic or surgical loss of a large mass of skeletal muscle tissue beyond the inherent regenerative capacity of the body, generally leading to a severe functional deficit^9^. Due to a significant loss of nerve and vascular supply accompanied by inflammation driven fibrosis, the self-repair process is not adequate to generate sufficient new muscle in time to prevent a chronic scar^10,11^. Although VML is widespread among the civilian population, military personnel in particular are more prone to such damage due to combat-related musculoskeletal injuries^12^. According to a recent study, 65% of soldiers who retired due to various injuries reported a muscle condition, while 92% of these cases included VML^13^.

Free functional muscle transfer (FFMT) is the preferred procedure to treat VML, which entails transfer of donor muscle along with nerve and blood vessels from another part of the body to the injury site to facilitate re-innervation and re-vascularization of the graft region^10,12,14^. Although FFMT remains the gold standard, its success is limited by donor site morbidity, long operative time, and prolonged de-innervation of motor end plates in the donor muscle^15^. As an alternative approach, tissue-engineered skeletal muscle constructs have been fabricated using scaffold-based as well as scaffold-less technologies. Decellularized extracellular matrix (dECM) remains the most prevalent scaffold material used for muscle reconstruction following VML injuries^16,17^. Although dECM-based scaffolds recapitulate the native ECM composition, their efficacy is challenged by their fast resorption rates in the body and failure to provide topographical guidance to regenerating myofibers thereby leading to randomly oriented fiber recruitment and subsequent scar tissue formation^18,19^. The ECM secreted by skeletal myofibers is arranged in a network of micro-nano fibrils, which are aligned along the myofibers. Aligned nanofiber-based scaffolds accurately replicate the topographical cues of native ECM architecture and has been reported to promote aligned myogenesis, cell migration, survival, and angiogenesis^20^.

Persistent inflammation, fibrosis, revascularization, and reinnervation remain the major impediments to complete recovery of contractile function following major skeletal muscle trauma like VML^21–25^. Hence, in addition to topographical guidance, tissue-engineered scaffolds must be myo-conductive, promote angiogenesis through either pre-vascularization or incorporation of vasculogenic accelerant, have immunomodulatory effect as well as facilitate reinnervation to overcome the pathophysiology of VML and achieve functional restoration. Direct administration of anti-inflammatory agents can reduce fibrosis but has been shown to hinder muscle regeneration^26,27^. Acellular scaffolds have been unable to address such multidimensional challenges to date and reinforces the necessity of incorporating multiple cell types in a scaffold^19^. Pre-vascularized constructs fabricated by coculture of myoblasts and endothelial cells on aligned nanofibrous scaffolds have been found to promote organized myofiber regeneration and vascular integration in murine VML model^28^. Although angiogenic and immunomodulatory strategies have been explored in VML repair, lack of innervation in an engineered muscle remains one of the major impediments to its success as a functional muscle replacement^29^. In the absence of innervation, the functional maturation of myofibers cannot proceed^30^. Hence it is imperative that tissue engineering strategies for muscle replacement should consider innervation as an essential component of the biofabrication process itself to facilitate maturation of myofibers in vitro as well as promote reinnervation of damaged host muscle in vivo following implantation in severe musculoskeletal injuries.

In the present study, we report the development of a novel pre-innervated tissue-engineered muscle construct for application in VML repair. Aligned nanofiber sheets were used to coculture skeletal myocytes and spinal motor neurons and explore the effect of innervation on maturation of myocytes in vitro. The bioscaffolds were further implanted in an athymic rat model of VML, evaluated for cell survival, effect on host muscle cross-sectional area (CSA), satellite cell proliferation, microvasculature, and neuromuscular junction density (NMJs) at acute time points of 1 and 3 weeks. Tissue engineering strategies for VML repair have been focused toward bulk muscle restoration and achieving re-innervation and re-vascularization of the implant. The present study provides a unique perspective by focusing on the pathophysiology of the remaining host muscle following VML. To our knowledge, this is the first report studying the acute effect of pre-innervated constructs on the regenerative microenvironment of injured host muscle following severe muscle trauma.

## Results

### Pre-innervation promotes myocyte fusion and NMJs in vitro

**Fig. 1:**
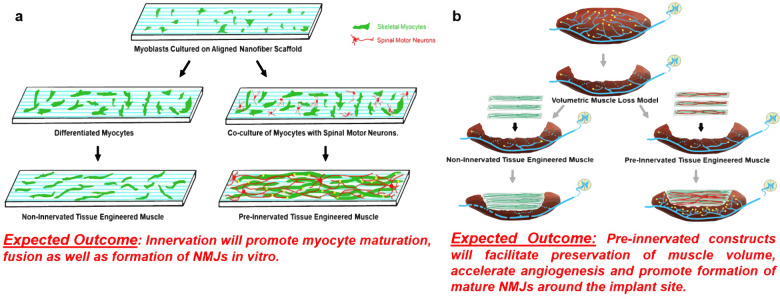
Concept of pre-innervated tissue-engineered muscle. The present study was focused on exploring the role of pre-innervation on myocytes in vitro and host neuromuscular environment in vivo following implantation. **a** For in vitro studies, our overarching hypothesis was that innervation would augment skeletal myocyte fusion, maturation, and formation of Neuromuscular Junctions (NMJs). **b** Volumetric Muscle Loss (VML) is defined as frank loss of muscle volume that is accompanied by chronic motor axotomy leading to denervation of the injured area. We used a standardized rat model of VML where >20% of the Tibialis Anterior (TA) muscle volume was resected to create a defect leading to potential damage to intramuscular branches of the host nerve and loss of motor end plates (or NMJs) near the injury area. For in vivo studies, our overarching hypothesis was that implantation of pre-innervated constructs would facilitate preservation of muscle volume, accelerate angiogenesis, enhance Acetylcholine Receptor (AchR) clustering and promote innervation of AchRs (mature NMJs) near the implant site at acute time point.

**Fig. 2:**
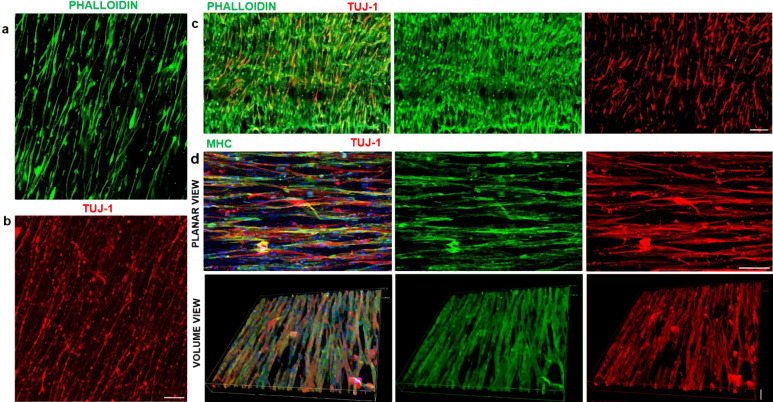
Motor neuron-myocyte culture on aligned nanofibers. **a**, **b** C2C12 Skeletal myocytes and spinal motor neurons were cultured separately as monocultures on aligned PCL nanofiber sheets to optimize media conditions and subsequently stained with Phalloidin and Tuj-1, respectively. Scale bar = 200 μm. **c** Subsequently, myocytes and motor neurons were cocultured on nanofiber sheets for optimizing coculture media. Scale bar = 200 μm. **d** In order, to confirm the formation of myotubes and neuromuscular bundles the motor neuron-myocyte cocultures on nanofiber sheets were stained for Myosin Heavy Chain (MHC) and Tuj-1. The neuromuscular bundles were visualized from compressed multiple Z-stack images in planar as well as volumetric view. Scale bar = 100 μm (Planar view), 70 μm (Volume view). The planar and volume view are from the same culture.

**Fig. 3:**
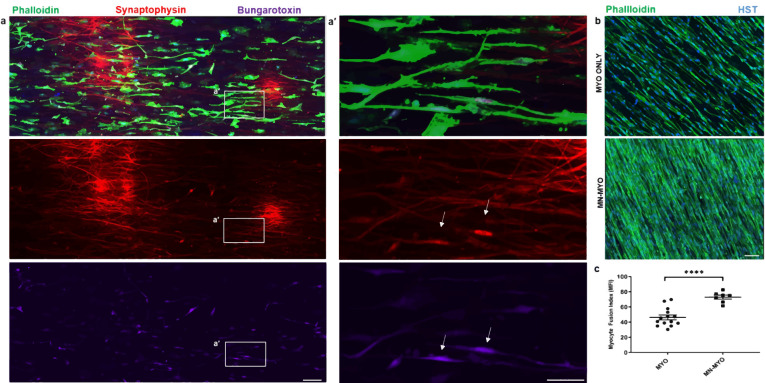
Innervation of myocytes and effect of motor neurons on myocyte maturation in vitro. **a** Rat spinal motor neurons were introduced on a bed of myofibers (Phalloidin) differentiated on aligned nanofiber sheet for 7 days and subsequently cocultured for another 7 days leading to innervation of the skeletal myofibers. Scale bar = 100 µm. **a′** Higher magnification view of the area marked by white box reveals structures colabelling for presynaptic marker (Synaptophysin) and Acetylcholine Receptor (AchR) clusters (Bungarotoxin) indicating formation of mature neuromuscular junctions in vitro (indicated by white arrows). Scale bar = 50 µm. **b** Myocytes exhibited greater fusion when cocultured with motor neurons (MN-MYO) as compared with monoculture (MYO). The cultures were stained with Phalloidin (myocytes) and nuclear marker Hoeschst (HST). Scale bar = 100 µm**. c** Myocyte Fusion Index (MFI) was calculated from multiple cultures (*n* ≥ 6), and coculture with motor neurons was found to significantly enhance MFI. For indicated comparison: *p* ≤ 0.0001 (****). Error bars represent standard error of mean.

Mouse skeletal myoblast cell line C2C12 were allowed to differentiate to form myotubes before coculturing with spinal motor neurons (Fig. 1a). The coculture system was initially optimized on tissue culture petri dish and monitored for spontaneous contraction of the myotubes (Supplementary Movies 1 and 2) before transitioning onto nanofiber sheets. Skeletal myocytes were grown on aligned polycaprolactone (PCL) nanofiber scaffolds and allowed to differentiate. Differentiated myofibers were found to align along the direction of nanofibers as observed by staining for F-actin (Fig. 2a). Similarly, spinal motor neurons cultured on the nanofibers exhibited axons aligning along the nanofiber orientation (Fig. 2b). Subsequently, both motor neurons and myocytes were cocultured on the nanofiber scaffolds. Motor neuron-myocyte coculture led to formation of intertwined neuromuscular structures aligned along the nanofibers (Fig. 2c, d). The neuromuscular constructs were found to form thick bundles when multiple Z-stack images were compressed and visualized in a 3D volumetric manner (Fig. 2d). Skeletal myocytes and spinal motor neurons were further confirmed by staining for Myosin Heavy Chain (MHC) protein (Fig. 2d) and Choline Acetyltransferase (ChAT) motor neuron marker, respectively (Supplementary Fig 1a). Within 7 days of coculture on the nanofiber scaffolds, NMJs were observed by colabelling for presynaptic marker Synaptophysin and Bungarotoxin mediated identification of post synaptic Acetylcholine Receptors (AchR) (Fig. 3a-a**′**). Motor neurons were also found to promote myocyte fusion in vitro leading to significantly higher myocyte fusion index (MFI) as compared to myocyte only cultures (Fig. 3b, c). Taken together, these data clearly demonstrate that innervation not only leads to NMJs in vitro but also facilitates myocyte fusion.

### Bioscaffold implantation in athymic rat model of VML

**Fig. 4:**
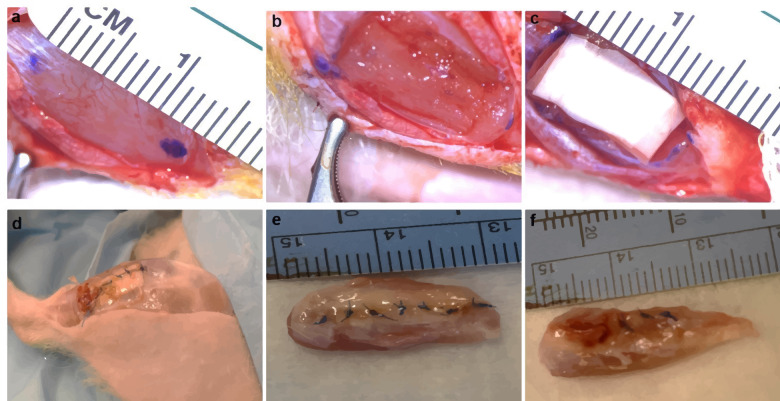
Bioscaffold implantation in VML model. **a**, **b** Surgical resection of TA muscle to create VML model in rats. **c**, **d** Implant of cell-laden nanofiber sheets in muscle defect. Scaffold and overlying fascia secured with sutures. **e**, **f** At terminal time points, animals were sacrificed, and TA muscle was excised. Nanofiber sheets were seen in **e.** Repair Group whereas injury site was recessed in **f** No Repair group.

The tibialis anterior (TA) muscle of athymic rats was exposed and a 10 mm × 7 mm × 3 mm (length × width × depth) segment of the muscle was excised corresponding to ~20% of gross muscle weight to create a VML model (Fig. 4a, b). Three pieces of cell laden or acellular nanofiber sheets each having dimensions 10mmx5mm were stacked and laid within the muscle gap (Fig. 1b). The animals were randomized into the following repair groups: (i) three stacked nanofibrous sheets (per animal) containing coculture of motor neurons and myocytes (MN-MYO; *n* = 6 for 1 and 3-week time points); (ii) three stacked nanofibrous sheets containing myocytes only (MYO; *n* = 4 for 1 week and *n* = 6 for 3-week time point); (iii) three stacked acellular nanofibrous sheets only (SHEETS; n = 3 for 1 week and 3-week time point), and (iv) NO REPAIR (*n* = 5 for 1 week and *n* = 3 for 3-week time point) (Fig. 4c). At terminal time point of 1 and 3 weeks post implant, the nanofiber sheets were visible upon TA exposure and appeared intact (Fig. 4d). Further, the graft area in the No Repair group appeared to be recessed and atrophied as compared to the Repair groups (Fig. 4e, f).

### Pre-innervated bioscaffolds maintain TA volume

**Fig. 5:**
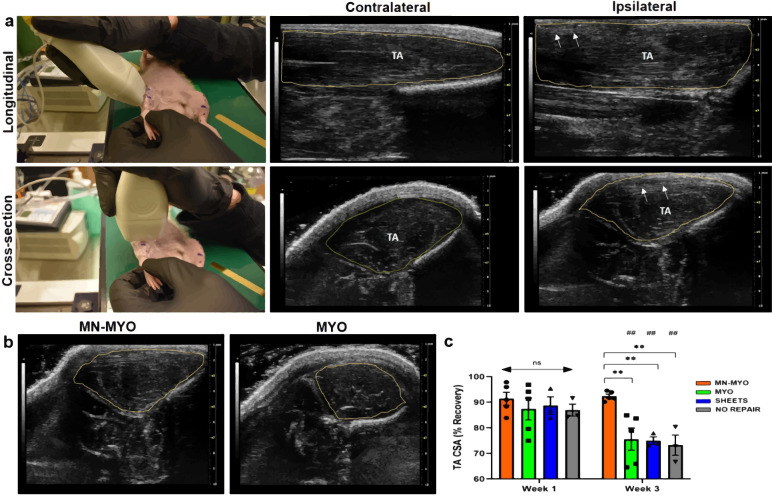
Pre-innervated bioscaffolds lead to increased recovery of muscle cross-sectional area following VML. **a** Representative images demonstrating cross-sectional and longitudinal ultrasound imaging of rat TA muscle. The TA region is demarcated by the yellow line. Multiple nanofiber sheets appeared to be stacked towards the edge of TA in both views as indicated by the white arrows. **b**, **c** TA cross-sectional area was measured for MN-MYO (*n* = 5 for week 1 and week 3), MYO (*n* = 5 for week 1 and week 3), Sheets (*n* = 3 for week 1 and week 3) and No Repair (*n* = 3 for week 1 and week 3) groups by drawing boundaries as indicated by yellow lines. **c** The recovery of TA CSA was calculated over time across all groups and expressed as percentage of respective contralateral TA CSA. Mean TA CSA percentage as compared to respective contralateral side were as follows – (Week 1) MN-MYO: 91.4; MYO: 87.3; Sheets: 88.7; No Repair: 86.9. (Week 3) MN-MYO: 92.3; MYO: 75.6; Sheets: 74.9; No Repair: 73.2. For indicated comparisons the individual *p* values were as follows:- MN-MYO vs MYO (Week 3) – *p* = 0.0033 (**); MN-MYO vs Sheets (Week 3) – *p* = 0.0089 (**); MN-MYO vs No Repair (Week 3) – *p* = 0.0037 (**), MYO (Week 1 vs Week 3) – *p* = 0.0014 (##), SHEETS (Week 1 vs Week 3) – *p* = 0.0033 (##) and No Repair (Week 1 vs Week 3) – *p* = 0.0034 (##). Error bars represent standard error of mean.

Ultrasound mediated imaging of the TA was performed at 1 and 3 weeks following surgery to monitor muscle health over time. The nanofiber sheets were observed as multiple stacked linear and hyperechoic structures close to the surface of the muscle and were visible up to 3 weeks post repair during longitudinal as well as cross-sectional imaging (Fig. 5a). Muscle regeneration was evaluated by comparing percent recovery measurements between the cross-sectional areas (CSA) of the ipsilateral and contralateral TA muscles per animal in each group at 1 and 3 weeks post surgery (Fig. 5b, c). There was no significant difference in percent TA CSA recovery after 1 week across all the groups. However, at 3 weeks, the MN-MYO group had significantly higher percent recovery of TA CSA as compared to the other groups. In addition, by 3-week time point, the MYO and Sheets only group exhibited significant reduction in TA CSA compared to 1-week time point (Fig. 5c). The results clearly demonstrate that pre-innervated constructs significantly enhance muscle CSA recovery within 3 weeks of VML injury.

### Evaluation of acute cell survival in bioscaffolds in vivo

**Fig. 6:**
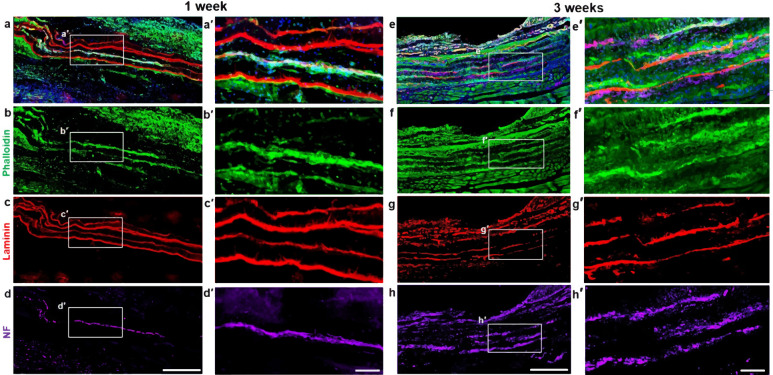
Cellular and morphological evaluation of pre-innervated bioscaffolds following implantation in a VML model. **a**–**h** Longitudinal sections near the repair site of animals implanted with nanofibers with motor neurons + myocytes (MN-MYO) at1 week (**a**–**d**) and 3 weeks (**e**–**h**) time point. The nanofiber sheets were coated with Laminin prior to culturing cells and hence the stacked sheets were identified based on Laminin stain (red). Scale bar = 500 µm. (**a′**–**h′**) Magnified view of the corresponding region inside the white box. Thick bundles of myocytes (Phalloidin: green) and motor axons (NF: purple) were observed within the stacked nanofiber sheets both at 1 week and 3 weeks’ time point. Scale bar = 100 µm.

All animals were sacrificed after 1 week or 3 weeks and the whole anterior muscle compartment of the hind limbs were fixed in paraformaldehyde. The muscles were cryopreserved, embedded in OCT, sectioned, and stained. Immunohistochemical analysis of longitudinal sections from the MN-MYO group revealed thick elongated myocytes stained with Phalloidin (F-actin) and motor axons stained with Neurofilament (NF) within the implanted nanofiber sheets (Fig. 6). The presence of motor neurons within the sheet was confirmed by staining with ChAT (Supplementary Fig 1b). Further, analysis of TA cross-sections in MN-MYO and MYO group showed presence of MHC^+^ myocytes within the implanted sheets after 3 weeks, although axonal NF staining was negative for the MYO group (Supplementary Fig. 2a, b). The nanofiber sheets were observed to be intact up to 3 weeks. These results indicate the survival of implanted motor neurons and myocytes at acute time point following a VML repair.

### Pre-innervated bioscaffolds increase satellite cell density

**Fig. 7:**
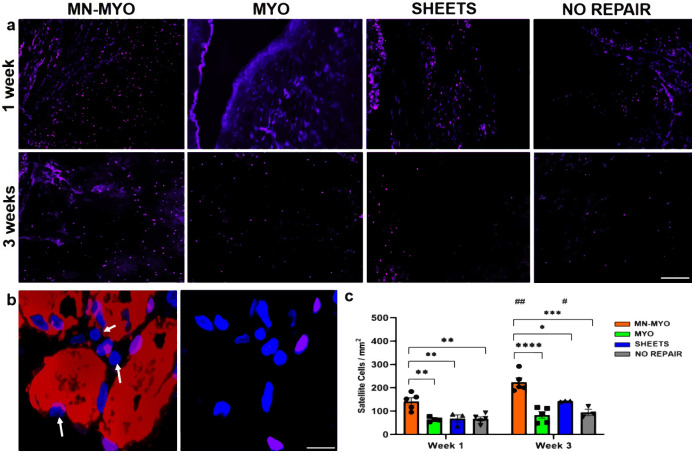
Pre-innervated bioscaffolds increase satellite cell density near injury area following VML. **a** Muscle satellite cells near the injury area were identified by staining for satellite cell marker – Pax 7 (Purple) across all the groups 1 week and 3 weeks following VML. Scale bar = 100 µm. **b** Representative image of a higher magnification view of satellite cells. Pax 7+ nuclei (Purple) located on the periphery of Skeletal Muscle Actin + (Red) myofiber and colabelling with pan-nuclear marker Hoeschst (Blue) were identified as satellite cells. Pax-7^−^/HST^+^ nuclei are indicated by white arrows. Scale bar = 10 µm. **c** Satellite cell density near the injury area (5 mm^2^) was counted across MN-MYO (*n* = 5 for week 1 and week 3), MYO (*n* = 4 for week 1 and *n* = 5 for week 3), Sheets (*n* = 3 for week 1 and week 3) and No Repair (*n* = 5 for week 1 and *n* = 3 for week 3) groups. Mean satellite cell density of each group were as follows: (week 1) MN-MYO - 141.4; MYO - 64.0; Sheets – 67.8; No Repair – 66.9; (week 3)) MN-MYO - 224; MYO – 83.2; Sheets – 142.7; No Repair – 94.7. For indicated comparisons the individual *p* values were as follows: (week 1) MN-MYO vs MYO – *p* = 0.0029 (**); MN-MYO vs Sheets – *p* = 0.0081 (**); MN-MYO vs No Repair – *p* = 0.0024 (**). (Week 3) MN-MYO vs MYO – *p* < 0.0001 (****); MN-MYO vs Sheets – *p* = 0.0202 (*); MN-MYO vs No Repair – *p* = 0.0006 (***). (Week 1 vs Week 3) MN-MYO – *p* = 0.0034 (##); Sheets – *p* = 0.034 (#). Error bars represent standard error of mean.

Satellite cells are resident myogenic precursor cells essential for muscle regeneration^31^. Activation and mobilization of satellite cells to the sites of injury is a major contributor to the regenerative capability of skeletal muscle^32^. Satellite cell density near the injury area was observed across all groups by staining with Pax7 (Fig. 7a). Pax7^+^ nuclei located on the periphery of Skeletal Muscle Actin^+^ myofibers and colabelling with pan-nuclear marker DAPI were identified as satellite cells (Fig. 7b). Importantly, the pre-innervated MN-MYO group exhibited significantly higher density of satellite cells near the injury area as compared to other groups at 1 and 3-week time points confirming that innervated constructs can trigger satellite cell recruitment thereby potentially facilitating muscle regeneration (Fig. 7c).

### Pre-innervated bioscaffolds promote revascularization

**Fig. 8:**
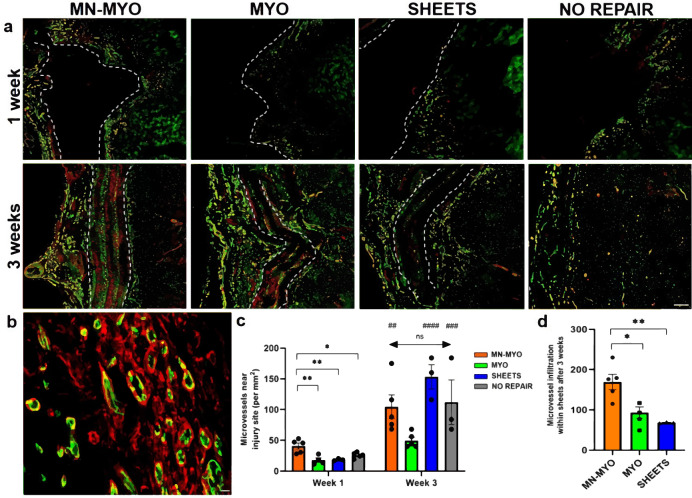
Pre-innervated bioscaffolds promote revascularization within and outside the injury area following VML. **a** Endothelial cells and microvasculature near the injury area were identified by staining for endothelial cell marker – CD31 (Green) and Smooth Muscle Actin (Red) across all groups 1 week and 3 weeks following VML. The implanted sheets are demarcated by broken white lines. Scale bar = 200 µm. **b** Representative image of a higher magnification view of endothelial cells and micro-vessels. Structures expressing CD31 (Green) and Smooth Muscle Actin (Red) with a visible lumen and an area > 50 µm^2^ were defined as micro-vessels. Scale bar = 10 µm. **c** Microvessel density near the injury area (5 mm^2^) was counted across MN-MYO (*n* = 5 for week 1 and week 3), MYO (n = 4 for week 1 and *n* = 5 for week 3), Sheets (*n* = 3 for week 1 and week 3) and No Repair (*n* = 5 for week 1 and *n* = 3 for week 3) groups. Mean microvessel density of each group were as follows: (Week 1) MN-MYO – 40.9; MYO – 17.7; Sheets – 18.4; No Repair – 25.8. (Week 3) MN-MYO – 116; MYO – 49.6; Sheets – 153.3; No Repair – 112. For indicated comparisons the individual *p* values were as follows: (Week 1) MN-MYO vs MYO – *p* = 0.0024 (**); MN-MYO vs Sheets – *p* = 0.0061 (**); MN-MYO vs No Repair – *p* = 0.0321 (*). (Week 1 vs Week 3) MN-MYO – p = 0.0049 (##); Sheets – *p* < 0.0001 (####). No Repair – *p* = 0.001 (###). Error bars represent standard error of mean. **d** Microvessel infiltration inside the implanted sheets was evaluated across the repair groups at 3-week time point. Mean number of microvessels inside the sheet region were as follows: MN-MYO – 169.2; MYO – 93; Sheets – 68.33. For indicated comparisons the individual *p* values were as follows: MN-MYO vs MYO – *p* = 0.0129 (*); MN-MYO vs Sheets – *p* = 0.0056 (**). Error bars represent standard error of mean.

Revascularization of the injured area/implant is critical for survival of implanted cells and integration with host vascular system. Vascularization near the injury area as well as within the implanted nanofiber sheets was evaluated by staining tissue sections with endothelial cell specific marker-CD31 and Smooth Muscle Actin (SMA) (Fig. 8). CD31^+^/SMA^+^ structures with a visible lumen and cross-sectional area greater than 50 µm^2^ were defined as microvessels (Fig. 8a, b). Although the MN-MYO group showed increased presence of microvessel-like structures adjacent to the injury site at 1-week time point, no significant difference in microvasculature near the injury site was observed across groups by 3 weeks. (Fig. 8c). In addition, none of the groups exhibited migration of endothelial cells within the implanted sheets by 1 week (Fig. 8a). However, revascularization of the implanted nanofiber sheets was observed across groups by 3 weeks following VML repair, with the MN-MYO group exhibiting the highest number by microvessels within the sheets (Fig. 8a, c). The results suggest that pre-innervated tissue-engineered muscle constructs can augment revascularization within as well as outside the injury area following VML repair.

### Effect of pre-innervated bioscaffolds on AchR expression

**Fig. 9:**
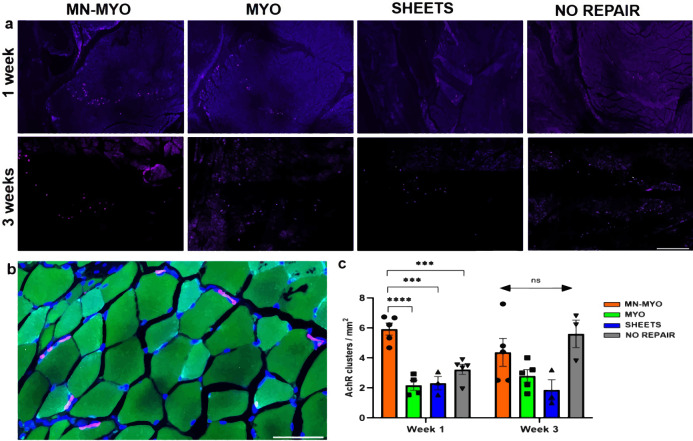
Acetylcholine receptor (AchR) clusters near injury area following VML. **a** AchR clusters near the injury area were identified by staining with Bungarotoxin (Purple) across all groups 1 week and 3 weeks following VML Scale bar =500 µm. **b** Representative image of a higher magnification view of pretzel shaped AchR clusters (Purple) on the periphery of muscle fibers (Phalloidin-488). Scale bar = 50 µm. **c** AchR cluster density near the injury area (5 mm^2^) was counted across MN-MYO (*n* = 5 for week 1 and week 3), MYO (*n* = 4 for week 1 and *n* = 5 for week 3), Sheets (*n* = 3 for week 1 and week 3) and No Repair (*n* = 5 for week 1 and *n* = 3 for week 3) groups. Mean AchR cluster density (per mm^2^) of each group were as follows: (Week 1) MN-MYO – 5.9; MYO – 2.2; Sheets – 2.3; No Repair – 3.2. (Week 3) MN-MYO – 4.4; MYO – 2.8; Sheets – 1.9; No Repair – 5.6. For indicated comparisons the individual *p* values were as follows: (Week 1) MN-MYO vs MYO – *p* < 0.0001 (****); MN-MYO vs Sheets – *p* = 0.0001 (***); MN-MYO vs No Repair – *p* = 0.0006 (***). Error bars represent standard error of mean.

Acetylcholine Receptor (AchR) clusters have major implications in formation and maintenance of motor end plates during muscle development as well as regeneration^33,34^. Indeed, staining with bungarotoxin, a known marker of nAchR α1 receptors in muscles^35^, showed the presence of pretzel-shaped AchR clusters around the injury area across all groups (Fig. 9a). A count of AchR clusters near the injury area revealed that the MN-MYO group had significantly more AchR clusters than the other groups at the 1 week time point (Fig. 9c). However, by 3 weeks following VML repair, although there was a sharp increase of AchR clusters in the NO REPAIR group as compared to 1 week (p = 0.0507), no statistically significant differences were observed in AchR cluster density among groups. Hence, although pre-innervation was found to enhance expression of AchRs at the 1-week time point, this effect relative to the other treatment groups was not maintained out to 3 weeks.

### Pre-innervated bioscaffolds promote NMJ formation in vivo

**Fig. 10:**
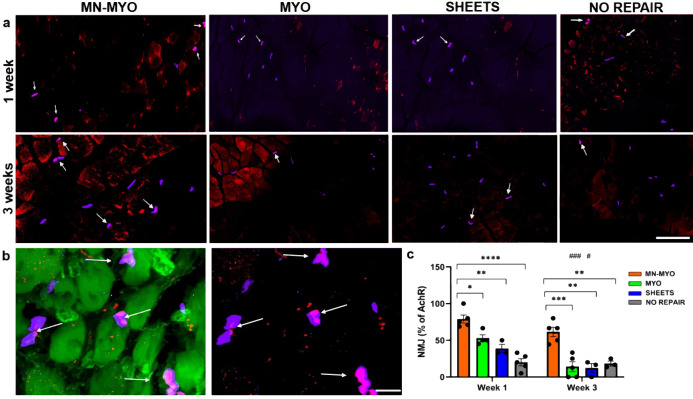
Pre-innervated bioscaffolds promotes formation of nmjs near injury area following VML. **a** NMJs near the injury area were identified by double staining with Bungarotoxin (Purple) and presynaptic marker Synaptophysin (Red) across all groups 1 week and 3 weeks following VML and are indicated by white arrows. Scale bar = 100 µm. **b** Representative image of a higher magnification view of mature NMJs (indicated by white arrows) comprising of pretzel shaped AchR clusters (Purple) colabelling with presynaptic marker Synaptophysin (Red) located on the periphery of muscle fibers (Phalloidin-488). Scale bar = 10 µm. **c** Percentage of AchR clusters near the injury area (5 mm^2^) that were innervated (Synaptophysin^+^) was counted across MN-MYO (*n* = 5 for week 1 and week 3), MYO (*n* = 4 for week 1 and *n* = 5 for week 3), Sheets (*n* = 3 for week 1 and week 3) and No Repair (*n* = 5 for week 1 and *n* = 3 for week 3) groups to depict maintenance/formation of NMJs in the host muscle. Mean percentage of NMJ of each group were as follows: (Week 1) MN-MYO – 78.9; MYO – 52.9; Sheets –38.7; No Repair – 20.2. (Week 3) MN-MYO – 61.5; MYO – 14.1; Sheets –12.2; No Repair – 18.3. For indicated comparisons the individual *p* values were as follows: (Week 1) MN-MYO vs MYO – p = 0.0168 (*); MN-MYO vs Sheets – *p* = 0.0012 (**); MN-MYO vs No Repair – p < 0.0001 (****). (Week 3) MN-MYO vs MYO – *p* = 0.0004 (***); MN-MYO vs Sheets – *p* = 0.0011 (**); MN-MYO vs No Repair – *p* = 0.0031 (**). (Week 1 vs Week 3) MYO – *p* = 0.0008 (###); Sheets – *p* = 0.0442 (#). Error bars represent standard error of mean.

Although AchR clustering is indicative of motor end plate health, they are not always the actual points of innervation, i.e. NMJs. NMJs are indicative of muscle health and their loss has been implicated in neuromuscular degeneration associated with inflammation, denervation, and atrophy^36^. Innervated AchRs or NMJs were identified as pretzel-shaped structures which were colabelled with presynaptic marker Synaptophysin and postsynaptic AchR marker (Bungarotoxin) (Fig. 10a, b). The percentage of AchR clusters near the injury area which were positive for Synaptophysin was calculated to quantify NMJs (Fig. 10c). Pre-innervated constructs (MN-MYO) were found to have significantly higher percentage of mature NMJs as compared to other groups at both 1 and 3 weeks following implantation in the VML model indicating the potential role of pre-innervation in enhancing formation of NMJs (Fig. 10c). Further, a significant decrease in innervated NMJs in the MYO and Sheets only groups was observed by 3 weeks following VML repair (Fig. 10c). This clearly demonstrates the crucial role of pre-innervated constructs in maintaining/promoting innervation of AchRs in the surrounding host muscle.

## Discussion

Severe musculoskeletal trauma like VML is accompanied by progressive motor axotomy over several weeks, leading to denervation of the injured muscle thereby severely limiting functional recovery^37^. Hence, appropriate somato-motor innervations remain one of the biggest challenges in fabricating a fully functional muscle. Apart from augmenting the re-innervation process, tissue engineering strategies need to provide accurate cellular alignment and enable bulk muscle replacement to compensate for loss of muscle volume following VML. Aligned nanofiber scaffolds are the preferred biomaterial for muscle reconstruction since they not only promote myofibers alignment but can also be stacked to provide bulk to the engineered tissue. Aligned nanofiber scaffolds have been shown to facilitate NMJ formation in vitro as well as promote alignment of regenerating myofibers in vivo as compared to randomly oriented nanofibers^20,28,38^.

Most aligned nanofiber scaffolds used to date for VML repair are comprised of decellularized ECM or collagen which are prone to faster degradation and do not possess optimal mechanical properties to support organized myofibril regeneration. In addition, synthetic polymer derived aligned nanofiber scaffolds are usually electropsun from polymer solutions thereby enabling scale-up and fabrication of custom-designed sheets to fit the exact dimensions of an injured muscle. Although synthetic polymer derived aligned nanofibrous scaffolds have been shown to promote formation of functional NMJs in vitro^38^, they are yet to be used as scaffolds for VML repair. To our knowledge, the present study is the first report on using synthetic polymer-based aligned nanofiber scaffolds in a rat VML model. We have used commercially available nanofiber sheets made of polycaprolactone (PCL)-which is an FDA approved slowly degrading, bioresorbable polymer^39^. These aligned PCL nanofiber sheets were used as scaffolds for 3D motor neuron-myocyte coculture.

Skeletal myocyte fusion results in post-mitotic multinucleated myofibers and is crucial during embryonic development as well as for adult muscle regeneration and maintenance^40,41^. Myocyte fusion results in increased myonuclear count and is usually measured by calculating the ratio of nuclei number in myocytes with 2 or more nuclei versus the total number of nuclei and is represented as the Myocyte Fusion Index (MFI)^40,42^. Scaffold alignment and presence of motor neurons have been previously reported to promote myocyte fusion in vitro leading to increased myonuclear count^8,38^. We also observed a similar effect where motor neurons cultured on pre-differentiated skeletal myocytes on aligned nanofibers led to formation of mature NMJs and promoted fusion of myocytes to form multinucleate myofibers (Fig. 3). This is in agreement with previous report, which describes that enhanced fusion and maturation of myocytes are only observed when the myocytes are allowed to fully differentiate before introduction of the motor neurons and the coculture is maintained subsequently in serum-free conditions^5^. Although several intracellular, extracellular, and membrane-associated proteins have been implicated in regulating myoblast fusion, the exact molecular mechanisms behind this phenomenon – especially how motor neurons augment fusion – are yet to be fully understood^41^.

To evaluate the in vivo potential of pre-innervated constructs as a reconstructive approach to VML, we used a standardized model of VML in the rat TA muscle^43^. Although different muscles like abdominal wall^44^, latissimus dorsi^45^ and quadriceps femoris^46,47^ have been used to create a VML grade critical muscle defect, the TA muscle remains the preferred choice of researchers due to ease of surgical access and measurable functional deficit following VML. Most tissue engineering strategies towards VML repair are evaluated in small animal models^48^. Cell-based approaches generally comprise of cell lines (mouse/human) or primary cells and hence are carried out in athymic animals to allow in vivo survival and maturation of the constructs^20,28,48,49^. VML has been reported to lead to over 73% motor axotomy within 1 week post injury without significant change in the number of damaged axons up to 21 days^37^. This indicates that 1 and 3 weeks post injury are appropriate acute time points to evaluate survival of implanted cells as well as study the effect of pre-innervated implants on host neuromuscular anatomy. We used T-cell deficient athymic rats to prevent immunogenic reaction to implanted mouse C2C12 cells and primary rat motor neurons.

Non-invasive imaging of musculoskeletal tissue is crucial for performing an objective longitudinal analysis of muscle volume and quality that can provide insight into the efficacy of novel therapeutic interventions for muscle trauma^50^. In the context of preclinical VML studies, computed tomography (CT) remains the prevalent imaging modality to monitor muscle volume^51^. However, in a clinical trauma setting like VML, retrospective analysis as well as the need to transfer a patient for CT imaging, limits accurate design of longitudinal studies^50^. In contrast, the use of ultrasound in VML injuries provides a rapid, non-invasive, and cost-effective approach for assessing skeletal muscle health. Although, ultrasound has been used in clinical studies of VML^52^, it is yet to be utilized in preclinical rodent VML models. Ultrasound allows both quantitative measurements, such as muscle cross-sectional area to determine percent maintenance and/or recovery, and qualitative measurements, such as echogenicity to evaluate muscle arrangement and integrity^53^. In this study, we utilized ultrasound B-mode imaging for monitoring muscle health and evaluating the integrity of the implanted nanofiber sheets. The nanofiber sheets were visualized as hyperechoic linear structures up to 3 weeks indicating their accurate implantation and maintenance of structural integrity (Fig. 5a). This is consistent with another study where ECM derived scaffolds in VML patients were visualized as hyperechoic structures by ultrasound^52^. CSA of the TA muscle was measured to determine percent muscle maintenance at 1 and 3 weeks post surgery. We observed a significant decrease in TA cross-section over time for animals with non-innervated constructs (Fig. 5b, c). Possible explanations for the significant decrease in muscle area at an acute time point may be attributed to inflammation, stress response, impaired microcirculation as well as denervation^54^. Pre-innervation was found to maintain muscle volume over 3 weeks (Fig. 5c). As previous studies have shown, a correlation exists between muscle mass and strength^50^. Based on initial results combined with the available literature, future experiments should consider examining functional outcomes and explore possible correlations with ultrasound measurements at chronic time points.

The aligned nanofiber sheets were highly porous (80% porosity) and our method of stacking three layers of sheets allowed exchange of nutrients and oxygen through blood perfusion thereby facilitating survival of the implanted motor neurons and myocytes. Subsequent immunohistological analysis of longitudinal sections of the muscle allowed visualization of multiple layers nanofiber sheets and confirmed the presence of long thick bundles of skeletal myocytes and motor axons on the nanofiber sheets confirming acute survival of the implanted cells (Fig. 6). We also observed significant loss of Laminin in the muscle fibers adjacent to the injury site (Supplementary Fig 2b). This can be attributed to the damaged basement membrane following severe musculoskeletal injuries like VML^18^. In order to achieve a comprehensive understanding of the acute effects of innervation on the regenerative milieu of an injured muscle, we proceeded to investigate the density of satellite cells, microvasculature, AchR clusters, and mature NMJs near the injury area.

The robust regenerative capacity of skeletal muscles can be largely attributed to the resident myogenic precursor cells called muscle satellite cells^31^. These satellite cells lie quiescent in between the basal lamina and sarcolemma and gets activated within a few days after an injury. Activated satellite cells then differentiate to form myoblasts which fuse together to form new skeletal muscle fiber^31^. Satellite cells can be reliably identified by paired box transcription factor Pax-7, which is expressed in both quiescent and activated stages^55^. Although pre-vascularized tissue-engineered constructs have been shown to promote satellite cell activation upon implantation in a mild muscle injury model, the effects of pre-innervation on host satellite cell population are yet to be addressed^56^. Separate studies indicate that various neurotrophic factors like NGF and BDNF play a critical role in modulating satellite cell response within an injured muscle^57,58^. For instance, exogenous treatment with BDNF was enough to recover the regenerative capacity of satellite cells in BDNF-deficient mice after skeletal muscle injury^57^. Spinal motor neurons used in the present study for fabrication of pre-innervated constructs have been shown to secrete BDNF^59^. This can potentially explain the presence of significantly more Pax-7+ satellite cells near the injury area in MN-MYO group having pre-innervated constructs comprising of motor neurons and myocytes after 1 and 3 weeks of VML repair (Fig. 7). However, unlike Czajka et al.’s report showing satellite cell migration within a pre-vascularized tissue-engineered construct within 3 days of implantation, we did not observe any Pax-7+ satellite cells within our constructs by 3 weeks^56^ (Fig. 7). This is likely due to the difference in models; VML presents a very different pathophysiology than does a mild incision injury.

Tissue engineering strategies for VML repair demands bulk muscle reconstruction. Inadequate re-vascularization remains one of the major challenges to engineer thick skeletal muscle limiting nutrient exchange and survival of implanted cells^24^. Pre-vascularized constructs comprising of preformed vascular networks have been shown to promote microvasculature, vascular perfusion of the graft, and inosculation with host vascular system thereby significantly improving muscle regeneration following VML^24,28,56^. Neurotrophic factors like NGF, BDNF, GDNF, NT-3 have been reported to enhance angiogenesis in different tissues like skin, heart, and cartilage through receptor mediated activation or recruitment of proangiogenic precursor cells^60–62^. Spinal motor neurons secrete BDNF, whereas astrocytes can express a range of neurotrophic factors^59,63^. It is to be noted that although we strive to obtain a pure motor neuron population, we have detected minimal glial cells in our motor neuron cultures. Interestingly, we observed that the pre-innervated constructs had significantly more microvasculature near the injury area at 1 week following implantation in a VML model (Fig. 8c). The density of microvasculature around the injury site continued to significantly rise for most of the groups over time up to 3 weeks. However, despite such increased microvasculature around the injury area at the 1 week time point there was no evidence of graft revascularization. Microvessel infiltration within the implanted sheets was observed by 3 weeks following VML repair with pre-innervation significantly augmenting the graft revascularization process (Fig. 8d). It is also noteworthy, that a recent study using 3D printed scaffolds with muscle and neural progenitor cells for VML repair did not report any significant effect of pre-innervation on graft revascularization^8^. This could be attributed to the use of immature neuronal population with limited or different cytokine release profile as compared to the mature spinal motor neuron cultures that we employed. Although the molecular mechanisms of how pre-innervated constructs promote vascularization are the scope of future studies, it is reasonable to postulate that neurotrophic factors from spinal cord-derived cell population (comprised predominantly of motor neurons, but also potentially glia) may have triggered this increased microvasculature.

Muscle nicotinic AchRs are pentameric structures that are dispersed along the basal membrane of myofibers (extra-junctional) during fetal stage and progressively redistribute to form localized (junctional) pretzel-shaped clusters on adult muscle^64^. AchR clustering plays a pivotal role in skeletal muscle function and regeneration through formation of stable motor end plates thereby effecting functional restoration following severe musculoskeletal trauma. Early physical rehabilitation involving exercise has been shown to benefit patients with VML in recovering muscle force and range of motions^52^. Similarly, rehabilitative exercise in conjunction with bioengineered constructs augments functional restoration in murine models of VML by promoting formation of AchR clusters and mature NMJs^65–68^. One of the key findings of the present study is that pre-innervated constructs augments AchR clustering and NMJ formation. This is reflected in our results which show MN-MYO group have significantly more AchR receptor clusters and mature NMJs within 1 week of implanting in a VML model (Figs. 9 and 10). Clustering of AchRs in skeletal muscles is mainly controlled by motor innervation through secretion of neural agrin by the motor neurons^34,69^. This may be a reason behind increased density of AchR clusters near the injury area 1 week following implantation of pre-innervated constructs (Fig. 9c). It has been shown that extensive motor axotomy of VML injured muscle has been observed by 3 weeks post injury^37^. Such extensive denervation often initiates a compensatory mechanism in the host muscle, leading to an increase in overall count of AchRs, a phenomenon referred to as denervation supersentivity^70^. In this context, it is noteworthy that the No Repair group exhibited a sharp increase in AchR receptor density by 3 week indicating extensive denervation, whereas there was an overall decrease in the pre-innervated MN-MYO group (Fig. 9c). Hence, in order to further investigate, we looked for innervated AchR clusters that would indicate formation of synapses and NMJs. The MN-MYO group was found to have significantly higher percentage of innervated AchR clusters as compared to other groups after 1 and 3 weeks of VML repair (Fig. 10c). This confirms that pre-innervated constructs reduce the effect of denervation supersensitivity following injury as well as promote innervation of host AchRs and formation of NMJs around the injury area which can potentially have a significant effect in augmenting functional restoration at more chronic time points.

Although our study demonstrates the potential of pre-innervated constructs in promoting a regenerative environment following VML, several limitations exist. First, this study was conducted in athymic rats lacking an intact thymus and functional T-cells and was terminated at an acute time point. We did not observe any significant immune response at 1 week time point but there was significant macrophage infiltration into the injury area across all groups by 3 weeks (Supplementary Fig 3). This is possibly due to the innate immune response which is usually intact in nude rats and is mediated by macrophages, neutrophils, mast cells, and natural killer cells. Second, we speculate about the possible role of various neurotrophic factors in facilitating a pro-regenerative microenvironment. However, it is likely that the physical presence of preformed motor axonal network plays a crucial role. Hence, more in-depth studies are necessary to elucidate the cellular/molecular mechanisms behind the observed effects of pre-innervation in VML repair. Third, although the benefits of preformed axonal networks for in vitro tissue engineering and acute host responses are apparent in our results, longer term effects of extraneous neurons on neuromuscular integration and functional recovery are unclear. To address these shortcomings, ongoing studies in our lab are looking at effects of pre-innervated constructs on functional recovery at more chronic time points.

Although the effect of pre-innervated implants on graft neural integration has been reported recently^8^, this is the first study, to our knowledge, to explore the implications of pre-innervation on the host regenerative microenvironment in a rat VML model. To our knowledge, this is also the first report on the use of synthetic polymer derived aligned nanofiber scaffolds as an implant in a VML model. Additionally, we have demonstrated the efficacy of ultrasound as a potential non-invasive imaging modality for conducting longitudinal analysis of musculoskeletal health in rodent models of VML. Our results indicate that pre-innervation promotes myocyte maturation in vitro, increased satellite cell density and vascularization in the injury area as well as facilitates formation of NMJs thereby providing a favorable regenerative microenvironment for neuromuscular regeneration following VML. We believe that these findings in skeletal muscle injury model would stimulate further research into developing pre-innervated tissue-engineered constructs for application in smooth muscle as well as cardiac tissue engineering. In future work, these nerve-muscle constructs may also be fabricated using cells derived from adult human stem cell sources (e.g., iPSCs), thereby making them translational as an autologous, personalized bioengineered construct. These pro-regenerative effects can potentially lead to enhanced functional neuromuscular regeneration following VML, thereby improving the levels of functional recovery following these devastating injuries.

## Methods

### Isolation and culture of rat spinal motor neurons

Motor neurons were harvested from the spinal cord of E16 Sprague Dawley rat embryos following previously described procedure^71,72^. All harvest procedures prior to dissociation were conducted on ice. Briefly, spinal cords were extracted from the pups and digested with 2.5% 10X trypsin diluted in 1 mL L-15 for 15 min at 37 ^o^C. The digested tissue was triturated multiple times with DNAse (1 mg/mL) and 4% BSA and centrifuged at 280 × *g* for 10 min to pool all the cell suspension. Subsequently, the cell suspension was subjected to Optiprep mediated density gradient centrifugation at 520 × *g* for 15 min to separate the motor neuron population. Following centrifugation, the supernatant was discarded, and cells were resuspended in motor neuron plating media consisting of glial conditioned media. Glial conditioned media was made as described earlier^71^ and supplemented with 37 ng/mL hydrocortisone, 2.2 µg/mL isobutylmethylxanthine, 10 ng/mL BDNF, 10 ng/mL CNTF, 10 ng/mL CT-1, 10 ng/mL GDNF, 2% B-27, 20 ng/mL NGF, 20 µM mitotic inhibitors, 2 mM L-glutamine, 417 ng/mL forskolin, 1 mM sodium pyruvate, 0.1 mM β- mercaptoethanol, 2.5 g/L glucose to make complete motor neuron plating media.

### Mouse skeletal myoblast cell line (C2C12) culture

C2C12 cell line was maintained in growth media comprising of DMEM-High Glucose, supplemented with 20%FBS and 1% PennStrep. The cells were allowed to reach 80% confluency before inducing differentiation through differentiation media comprising of DMEM-High Glucose supplemented with 2% NHS and 1% Penicillin-Streptomycin.

### Fabrication of pre-innervated tissue-engineered muscle

A 15 cm × 15 cm PCL aligned nanofiber sheet was custom fabricated and purchased from Nanofiber Solutions LLC (Ohio, USA). The sheets were cut into 10mmx5mm pieces, placed in 24 well tissue culture plates and UV sterilized prior to coating with 20 µg/mL poly-D-lysine (PDL) in sterile cell culture water overnight. The sheets were subsequently washed thrice with PBS before coating with 20 µg/mL mouse laminin (Corning,354232) for 2 h. Pre-differentiated C2C12 cells were plated on the nanofiber sheets at a concentration of 2 × 10^5^ cells/sheet in growth media for 24 h before being cultured using differentiation media for 7 days in vitro (DIV) with regular changes of media. Dissociated motor neurons were plated on top of the myocyte layer at a concentration of 1 × 10^5^cells/sheet and the coculture was maintained with serum-free motor neuron media up to 14DIV with regular changes of media. The sheets with only myocytes were also kept on serum-free motor neuron media between 7-14DIV to maintain parity of cell culture condition between groups.

### Immunofluorescence staining of cell-laden nanofiber sheets

Samples were fixed for 35 min in 4% paraformaldehyde (EMS, Cat# 15710), washed three times with 1× PBS, and permeabilized in 0.3% Triton-X100 + 4% Normal Horse Serum (NHS) (Sigma) for 60 min. Samples were blocked in 4% NHS (Sigma) and all subsequent steps were performed using 4% NHS for antibody dilutions. For staining of actin and AchR, samples were incubated with Alexfluor-488-conjugated phalloidin (1:200, Invitrogen, A12379) and AlexaFluor-647-conjugated bungarotoxin (1:250, Invitrogen, B35450). For assessment of motor neuron morphology and maturity, separate fixed samples were incubated with an axonal marker Tuj-1 (1:250, Abcam, ab18207) and presynaptic marker Synaptophysin (1:500, abcam, ab32127) for 16 h at 4 °C followed by Alexa Fluor-568 antibody (Life Technologies). Images were acquired using a Nikon Eclipse TI A1RSI laser scanning confocal microscope.

### Quantification of myocyte fusion index

Multiple replicates of MN-MYO (*n* = 7) and MYO only (*n* = 14) cultures were considered for measuring myocyte fusion index (MFI) as per the following equation:

$${\mathrm{MFI}} = \frac{{{\mathrm{Number}}\,{\mathrm{of}}\,{\mathrm{nuclei}}\,{\mathrm{in}}\,{\mathrm{myocytes}}\,{\mathrm{with}}\,{\mathrm{more}}\,{\mathrm{than}}\,3\,{\mathrm{nuclei}}}}{{{\mathrm{Total}}\,{\mathrm{number}}\,{\mathrm{of}}\,{\mathrm{nuclei}}\,{\mathrm{within}}\,{\mathrm{myocytes}}}}$$

Individual myofibers were discerned and their margins marked based on staining with Phalloidin before counting the number of nuclei in each myofiber. At least three 2 mm^2^ area was considered per sample for counting MFI and the average was plotted for each sample (Fig. 3c).

### Bioscaffold implantation in rat model of VML

All procedures were approved by the Institutional Animal Care and Use Committee at the Michael J. Crescenz VA Medical Center and adhered to guidelines established in the PHS Policy on Humane Care and Use of Laboratory Animals (2015). Rats had access to food and water ad libitum and were pair-housed in a colony with a 12 hr light/dark cycle.

Adult male athymic rats (RNU strain 316; Charles River Labs) weighing 280-300 g were used as subjects for this study. All procedures were carried out under aseptic conditions while the animal was under general anesthesia (1.5–2% isoflurane, 1.5 L O2) and thermal support was provided via temperature-controlled water pad. After shaving the hair of the lower-left hind limb and applying a liberal coat of betadine solution, 0.25 mg of bupivacaine was administered subcutaneously along the planned incision line. Following a previously outlined procedure^43^, a longitudinal skin incision was made along the lateral aspect of the lower leg; care was taken not to cut through the underlying fascia covering the tibialis anterior (TA) muscle. The skin was bluntly dissected from the fascia along the length of the TA. A longitudinal incision (~1.5 cm) was made in the overlying fascia and the fascia was then gently dissected from the underlying TA muscle using a blunt probe, keeping the fascia intact for later repair. Once the muscle was exposed, a flat spatula was inserted between the tibial bone and TA muscle in order to isolate the TA/extensor digitorum longus (EDL) complex for further surgical manipulation. A mark was made 0.5 cm from the tibial tuberosity, indicating the proximal incision in the TA. A second mark was made 1.0 cm distal to the first and a 1.0 cm × 0.7 cm area was outlined on the TA using a surgical caliper (Fine Science Tools, cat # 18000-35). A 3 cm deep incision was made through the muscle at the proximal line and the scalpel turned parallel to the tibial bone to make a smooth cut through the muscle while following the outlined rectangle. Care was taken to avoid cutting completely through the TA or slicing the underlying EDL muscle. Once the portion of the muscle was removed, it was weighed and discarded. Deficits were repaired with three stacked cell-laden sheets (MN-MYO, MYO), three stacked acellular sheets alone (SHEETS), or were not repaired (NO REPAIR). Prior to implantation, the sheets were washed thoroughly with PBS to remove any leftover media. The fascia, connective tissue, and skin were closed in layers with 8-0 prolene, 6-0 prolene, or staples, respectively. At the conclusion of the surgery, the area was cleaned with alcohol and animals were given a subcutaneous injection of sustained-release meloxicam (4 mg/kg). Animals were placed on heating pads until recovered and returned to home cages.

### Ultrasound imaging of TA muscle following VML repair

Ultrasound images of the TA muscle were taken in both longitudinal and sagittal planes after 1- and 3-weeks post surgery, collecting both quantitative and qualitative data. Under isoflurane anesthesia, the hind limbs were shaved and depilation cream (Nair, Church & Dwight Co.) was applied to remove any remaining hair. With the rat in a supine position, the patella and malleolus were identified and ultrasonic gel (Aquasonic 100, Parker Labs) was applied to the TA region. Securing the ankle joint the transducer was held perpendicular to the TA muscle at its thickest point and cross-sectional images were obtained for 2 D area analysis, as shown in Fig. 5a. Longitudinal images were also recorded by holding the transducer parallel to the identified TA region between the patella and malleolus. All images were recorded under the same acquisition settings bilaterally in B-mode using the Vevo2100 imaging system equipped with a 32–56 MHz linear transducer (MS550S, FUJIFILM VisualSonics) and analyzed under blinded conditions using freely available Fiji software.

### Immunohistological assessment of injury site

Freshly harvested whole anterior muscle samples (TA + EDL) were fixed in 4% paraformaldehyde (EMS, Cat# 15710), submerged in 20% sucrose in 1X phosphate-buffered saline (PBS, pH 7.4) for density equilibration, frozen and cryosectioned axially and longitudinally (20 μm) across the middle portion of the graft region. Prior to staining, sections were washed three times in 1X PBS, blocked and permeablized in 4% normal horse serum (Sigma, G6767) with 0.3% Triton X-100 (Sigma, T8787) in 1X PBS for 1 h. All subsequent steps were performed using blocking solution for antibody dilutions. For staining of skeletal muscle actin, samples were incubated with rabbit-anti-skeletal muscle actin (1:500, abcam, ab46805) or mouse-anti-myosin heavy chain (1:10, Novus Biologicals, MAB4470) overnight at 4 °C followed by AlexaFluor-568 antibody (1:500, Invitrogen, A10042 and A10037) for 2 h at room temperature. Alternatively, for staining of actin, samples were incubated with AlexaFluor-488-conjugated phalloidin (1:400, Invitrogen, A12379) for 2 h at room temperature. For microvasculature staining, smooth muscle actin and endothelial cells were targeted, samples were incubated with mouse-anti-smooth muscle actin (1:500, abcam, ab7817) or rabbit-anti-CD31/PECAM1 (1:500, Novus, NB100-2284) overnight at 4 °C followed by AlexaFluor-568 antibody (1:500) or AlexaFluor-568 antibody (1:500, Invitrogen, A10087), respectively, for 2 h at room temperature. For staining of satellite cells, samples were incubated with mouse-anti-Pax7 (1:10, DSHB) overnight at 4°C followed by AlexaFluor-647 antibody (1:500, Invitrogen, A31573) for 2 h at room temperature. For staining of axons, samples were incubated with rabbit-anti-Neurofilament (1:500, abcam, ab8135) or mouse-anti-Neuroflament (1:500, BioLegend, 835601) overnight at 4°C followed by AlexaFluor-568 (1:500, Invitrogen, A21099), AlexaFluor-647 antibody (1:500, Invitrogen, A31573 and A31571) respectively, for 2 h at room temperature. For staining of laminin, samples were incubated with rabbit-anti-laminin (1:500, abcam, ab11575) overnight at 4°C followed by AlexaFluor-568 antibody (1:500) for 2 h at room temperature. For staining of neuromuscular junctions, samples were co-labeled with synaptophysin and bungarotoxin. Samples were incubated overnight with rabbit-anti-synaptophyhsin (1:500, abcam, ab32127) at 4 °C followed by AlexaFluor-568 antibody (1:500) and concurrently with AlexaFluor-647-conjugated bungarotoxin (1:1000, Invitrogen, B35450) for 2 h at room temperature. For staining of cell nuclei, samples were incubated with Hoescht (1:10,000, Invitrogen, H3570) for 20 min at room temperature. Secondary only staining was performed for each combination as negative control. Images were acquired using a Nikon Eclipse TI A1RSI laser scanning confocal microscope keeping the acquisition laser parameters same within each combination.

For quantitative measurement of satellite cell, micro-vessel, AchR cluster (10–40 clusters/sample), and mature NMJ density (10–40 NMJs/sample), an area of 5 mm^2^ (5 mm long and 1 mm wide) was chosen at 100 µm from injury/implant site towards the host muscle and defined as the injury area (Supplementary Fig 2c). At least three cross-sections each separated by 300 µm was considered for counting and average density was plotted in the graph and compared across groups.

### Statistics and reproducibility

All quantifications reported in this study were performed by personnel blinded to the treatment groups. All statistical analyses were performed using GraphPad PRISM software. For comparison between two groups only (Fig. 3c), an unpaired two-tailed Student’s t-test with Welch’s correction was used. For comparison between multiple groups during a single time point, a one-way analysis of variance (ANOVA) was performed with post hoc Tukey’s adjustment with 95% Confidence Interval (Figs. 7c, 8c, d, 9c, 10c). Significance was taken at *p* ≤ 0.05 (*), *p* ≤ 0.01 (**), *p* ≤ 0.001 (***), and *p* ≤ 0.0001 (****). A two-way mixed model ANOVA was performed with post hoc Sidak multiple comparison test to evaluate the effect of time (week 1 vs week 3) for each treatment and no repair groups. Significance was taken at *p* ≤ 0.05 (#), *p* ≤ 0.01 (##), *p* ≤ 0.001 (###), and *p* ≤ 0.0001 (####). All graphs were made in GraphPad PRISM and display mean ± standard error of mean (SEM).

### Reporting summary

Further information on research design is available in the Nature Research Reporting Summary linked to this article.

## Supplementary information

Supplementary Information

Supplementary Movie 1

Supplementary Movie 2

Description of Additional Supplementary Files

Reporting Summary

## Data Availability

Source data are provided with this paper. Remaining data are available from the corresponding author upon reasonable request.

## Source data

Source Data
Peer Review File
