# Supplementary material for: Pre-innervated tissue-engineered muscle promotes a pro-regenerative microenvironment following volumetric muscle loss
Source: Commun Biol. 2020 Jun 25;3:330. doi: 10.1038/s42003-020-1056-4 (PMC7316777; doi:10.1038/s42003-020-1056-4)
Supplement: Supplementary file 1 — Supplementary Information [file 42003_2020_1056_MOESM1_ESM.pdf]

Supplementary Materials

Pre-Innervated Tissue Engineered Muscle Promotes a Pro-Regenerative Microenvironment Following Volumetric Muscle Loss

Supplementary Figures

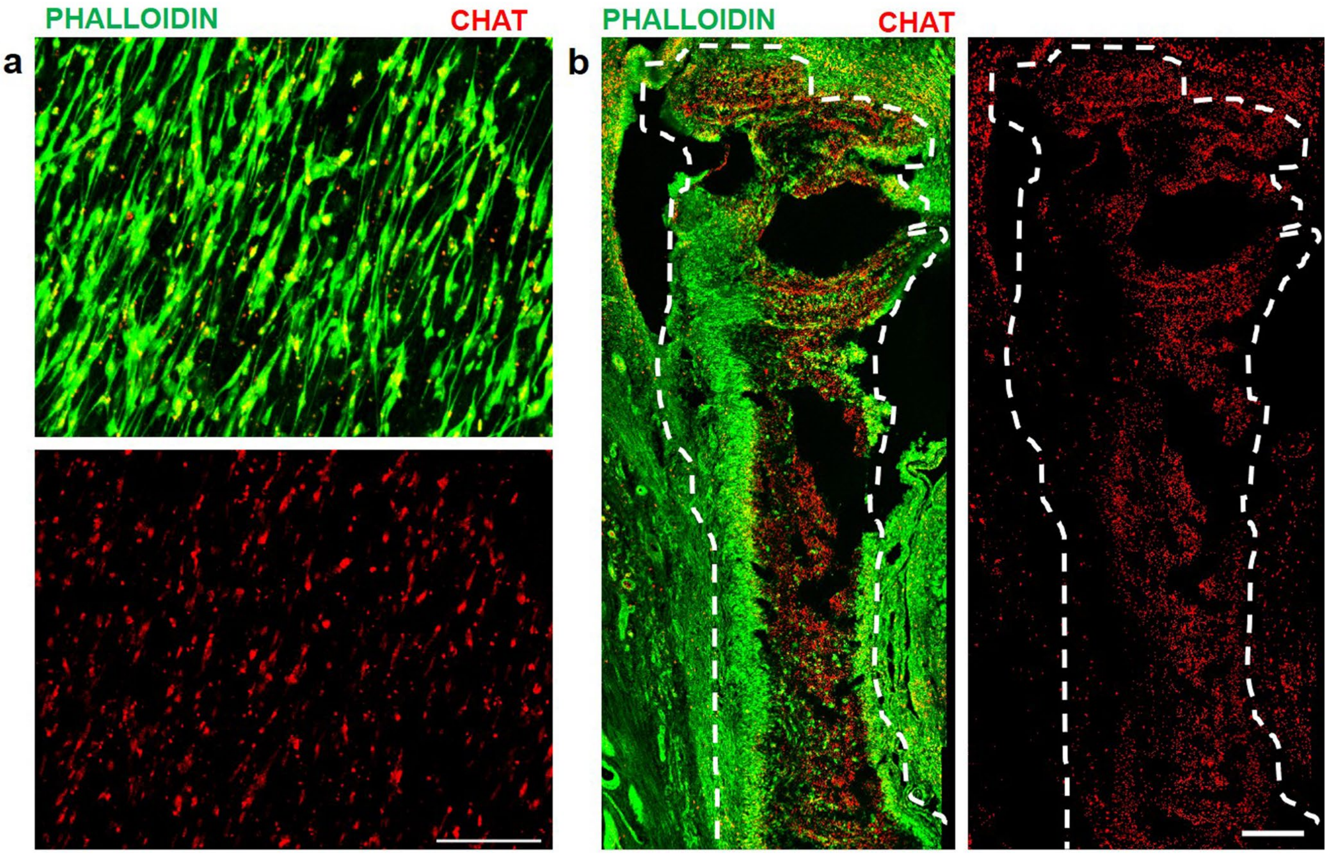

Supplementary Figure 1: In vitro and In vivo ChAT Expression by Motor Neurons. A) Motor neurons cocultured with skeletal myocytes on aligned nanofiber sheets were found to express mature motor neuron marker Choline Acetyl Transferase (ChAT) before being implanted. Scale bar = 200μm. B) The implanted motor neurons were found to express ChAT in vivo in and around the sheet region (broken white lines). Scale bar = 200μm.

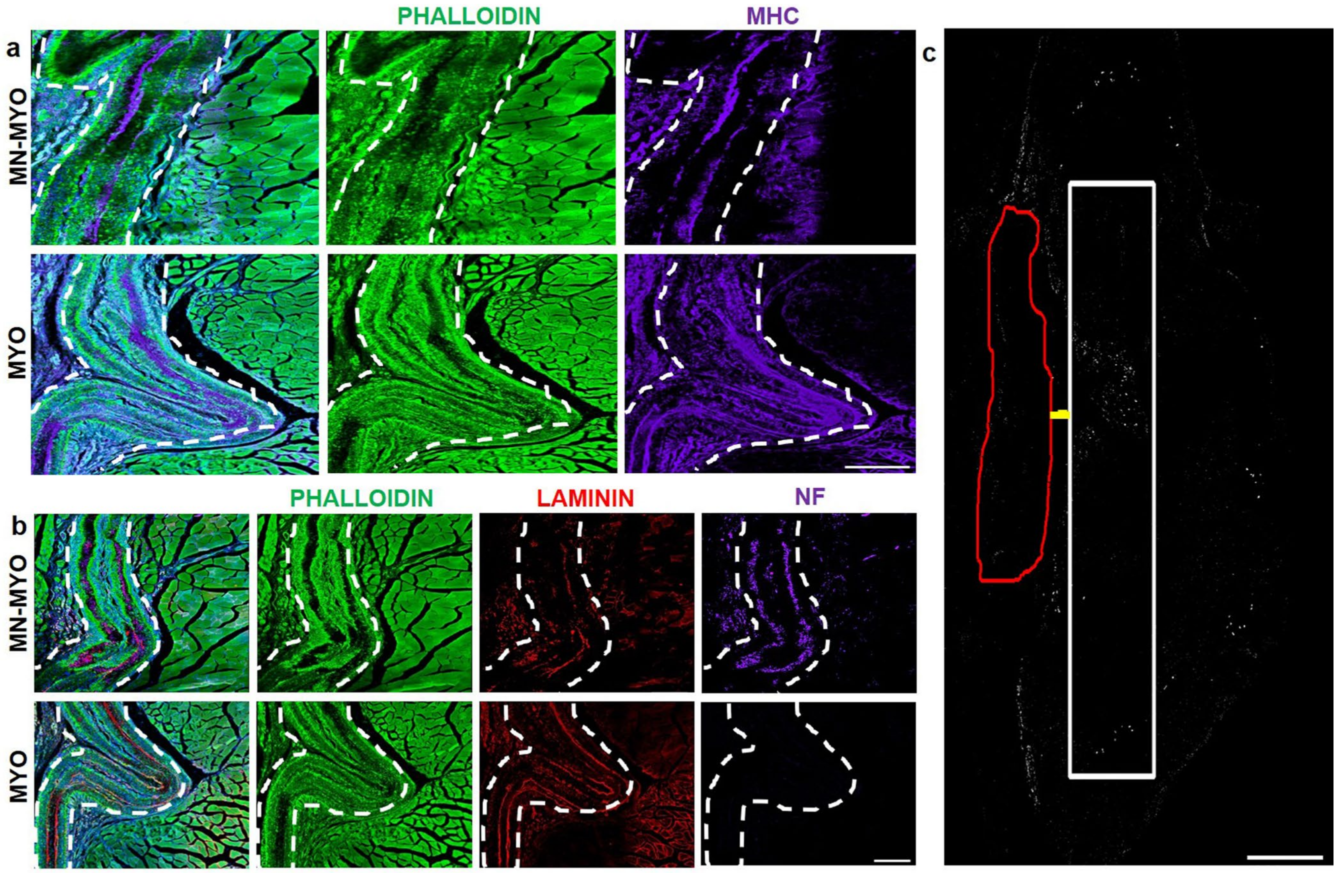

Supplementary Figure 2: Cross-Sectional Images of TA Muscle 3 Weeks Following VML Repair. a-b) Survival of myocytes and motor neurons inside the implanted nanofiber sheets were evaluated by staining cross-sections of the TA muscle with Myosin Heavy Chain (MHC) for myocytes and Neurofilament (NF) for motor neurons. The sheets are marked by broken white lines. Scale bar = 200μm **c**) An example of a cross-sectional image showing the injury area (red line) and a representative region of interest (white rectangular area of $5mm^2$) beginning at 100μm (yellow line) from the injury site was used for quantification purposes. White dots represent AchR clusters stained with Bungarotoxin. Scale bar = 1000μm.

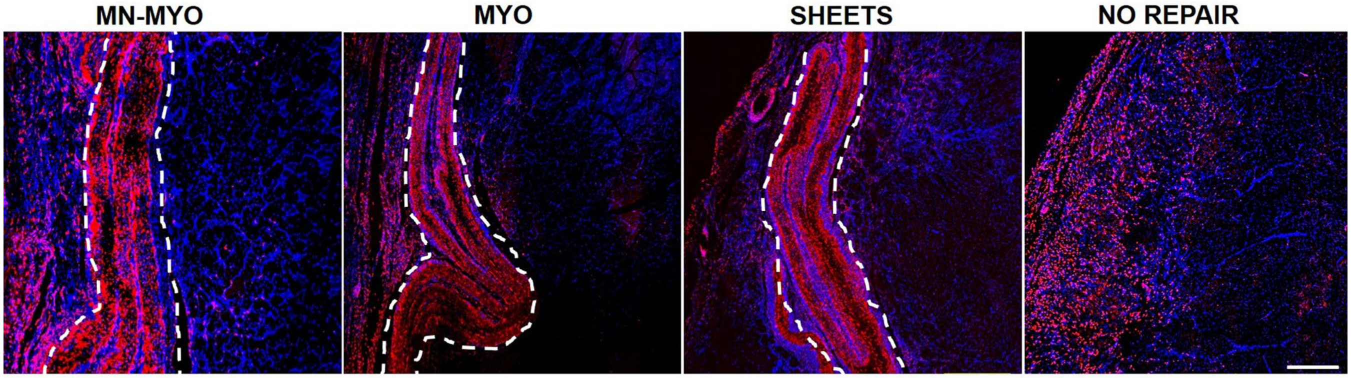

Supplementary Figure 3: Immune Reaction 3 Weeks Following VML Repair. Cross-sections of the TA muscle were stained with pan macrophage marker CD68 (red) and Hoeschst (blue) 3 weeks following VML repair. The implanted sheet area is demarcated by broken white lines. Scale bar = 500μm.
