# Supplementary material for: Pre-innervated tissue-engineered muscle promotes a pro-regenerative microenvironment following volumetric muscle loss
Source: Commun Biol. 2020 Jun 25;3:330. doi: 10.1038/s42003-020-1056-4 (PMC7316777; doi:10.1038/s42003-020-1056-4)
Supplement: Supplementary file 4 — Description of Additional Supplementary Files [file 42003_2020_1056_MOESM4_ESM.pdf]

Description of Additional Supplementary Files

File Name: **Supplementary Movie 1**
Description: A motor neuron aggregate (dark region) innervating myofibers and spontaneous contraction of myotubes

File Name: **Supplementary Movie 2**
Description: Motor neuron-myocyte coculture showing a single myotube spontaneously contracting.
