# Supplementary material for: Pre-innervated tissue-engineered muscle promotes a pro-regenerative microenvironment following volumetric muscle loss
Source: Commun Biol. 2020 Jun 25;3:330. doi: 10.1038/s42003-020-1056-4 (PMC7316777; doi:10.1038/s42003-020-1056-4)
Supplement: Supplementary file 7 — Peer Review File [file 42003_2020_1056_MOESM7_ESM.pdf]

Reviewers' comments:

Reviewer #1 (Remarks to the Author):

The authors have submitted a well-written manuscript that assesses the effect of implanted neuromuscular constructs on the acute regenerative response after VML injury. The constructs were composed of an aligned nanofiber scaffold, C2C12 muscle progenitors, and rat spinal cord motor neurons. The constructs were implanted into an athymic rat TA muscle VML model. The authors report an improvement of in vitro parameters of muscle fiber development and formation of NMJs, which are thought to support further observations in vivo of implanted cell survival and improved vascularization, satellite cell presence, and NMJ formation within the remaining tissue in close vicinity to the implant. Unfortunately, the manuscript only includes a subacute time point (7d post injury) and does not demonstrate a physiological effect of the reported observations. Without this determination it is difficult to understand the significance of the proposed findings, which are based solely on histological analyses that bear inherent sampling limitations. Moreover, the observations are nuanced, in that the observations of increased satellite cell, vascularization, and NMJ formation are observed in a region spatially distinct from the implanted material, which is a very different from the purpose/mechanism proposed in the introduction. At the same time, the defect area is devoid of these regenerated structures and there is no direct evidence of cell survival, in particular myocyte/fiber presence in the defect area. Please see the specific comments listed below.

Specific Comments:
1. Introduction 3rd Paragraph: please clarify what “random fiber recruitment” is.
2. Introduction Final Paragraph: When describing the study, there should be better distinction regarding the expected nature of satellite cell, vasculature, NMJ observations. Specifically, the graphical abstract seems to hypothesize that the changes are expected to occur in the area near the defect and not with the implanted material. This is mostly at odds with the bulk of the introduction that speaks to implanting a construct that will directly promote muscle fiber regeneration. Though unstated, the approach seems to focus more on implanting a construct that improves the survival or repair of the muscle tissue that is left after the initial injury.
3. Figure 2/Co-culture: Did you observe spontaneous contraction of the myotubes after differentiation? The terminology using bundles requires definition and clarity. The data do not support the formation of muscle fibers and certainly not bundles of fibers. Within the figure, please consider having panels a and b aligned in the same direction. Are these panels imaged at different magnification- the scale bars are different sizes?
4. Figure 3. The imaging appears over-exposed and does not allow observation of structural detail. Did the authors verify the phalloidin stain with a sarcomeric actin? Why is the density of cells in panel b for MN-MYO greater than that in panel a? For the fusion index, how were individual myotubes discerned to make the analysis? For panel b, please include labels for the staining like in panel a.
5. Figure 5. The sizing of the imaging should be consistent across groups in this figure and throughout the paper. Panel a: there appears little phalloidin staining within the defect raising question regarding the survival of implanted cells. Does the first image have four colors, with a blue channel for nuclei? If so, the lack of nuclei within the defect does no support cell survival. Panel b: The defect has greater phalloidin stain than the co-culture- this is not commented on the results. Additionally, in panels a and be there appears to be fibrous encapsulation that warrants discussion. Panel c: is concerning because it would seem that the muscle nearby should still show positive staining for phalloidin, as in panel b and even panel d. The lack of this positive staining precludes confident analysis of these findings.
6. Figure 6. The laminin staining is perplexing, in that it is unclear why the staining did not also surround muscle fibers in the remaining muscle and only stains the layers of the implanted scaffolds. In the methods, please specify the type of laminin that was used to coat the scaffold. The images are overexposed and do not allow visualization of structure. There are no structures in the images that are

obviously muscle fibers.
7. Figure 7. The analysis area cannot be estimated as it is unclear where the defect is. The panels appear to be taken at different magnification, specifically panel B appears at higher mag than the others? Panel e, the skeletal muscle actin stain is blown out and e' does not appear to show the dapi or hoescht stain for pax7 negative nuclei (?). Again, please provide similar sized images for comparison of like data.
8. Figure 8. The analysis is described as occurring 100 micron from the edge of the defect. Again, the images are of different mags making the quick comparison difficult. The error bars are 100 micron. Visualization of this distance reveals little to no vessels in any of the groups. Greater methodological clarity is need to assess what is quantified and compared.
9. Figure 9. Please provide similar size and mag images. From the images, it is not possible to know where the defect is and to ascertain where 100 micron from the defect edge is. Thus, it is not possible to know what is counted for the analysis.
10. Figure 10. Please provide similar size and mag images. The images do not support the observation of motor endplates with pretzel like morphology.
11. The methods need to include a detailed description of staining control studies performed and imaging procedures to control for intensity etc.

Reviewer #2 (Remarks to the Author):

Drs. Das and Cullen et al present a new method of combining nerve and muscle cells on a biomimetic scaffold to improve recovery from VML. This innovative new method builds upon prior research that has pre-seeded scaffolds with myocytes, endothelial or stem cells but tries to create more mature innervated scaffolds prior to implantation with combined myocyte and motor neuron transplants. While overall this is an intriguing new method, several concerns remain about the current version of the paper.
1) In Fig. 5, the group evaluates ChAT and NF-200 in vivo but do not stain for this in vitro. It is difficult to interpret the meaning of these stains within the sheets (are they increasing in number or decreasing once implanted) without baseline numbers from in vitro staining. In addition, quantification of these stain would be necessary to determine significant changes.
2) In Fig 6, it is difficult to determine if the stains point to survival of pre-implant MN-MYO group given the proper negative controls are not shown in the figure or as supplements to determine qualitative differences.
3) The paper would be strengthened by analysis at longer duration (typically at least 21 days in the muscle literature). This later time point is especially important given the point mentioned in the discussion about satellite cells and lack of their presence on the scaffold. Since these cells have been present on other scaffolds at these early time point, a later point would demonstrate if eventually these important components of muscular regeneration eventually do migrate onto the scaffold.
4) In the discussion, the authors mention that no signs of inflammation based on gross pathology were observed. To consider this point fully, further stains (such as H&E or specific inflammatory antibody staining) to evaluate for routine inflammatory response should be included. Otherwise, it is difficult to conclude the inflammatory response to the implant and this should be removed from the discussion.
Minor point: A reference for prior validation of MFI should be included in the method section.

Reviewer #3 (Remarks to the Author):

The manuscript by Das et al is an interesting study, in which the authors have created a novel bioengineered construct combining myoblasts and motor neurons for implantation into an established VML defect. This study addresses a current gap in bioengineered VML strategies which often lack focus on re-innervation of the critically injured muscle.
However, there are several issues that need to be addressed before being considered for publication.

Major Concerns
1. The goal or the hypothesis of the study is not clearly stated. As all analyses were done on tissue proximal to the implant, rather than within the implant itself, it is unclear whether the purpose of the implant was to directly integrate with the host muscle or to act as a source of positive paracrine signaling.

2. The majority of the analyses could have been done with congenic rats.
Myoblast can be isolated from rats and embedded in the construct along with motor neurons and then construct can be implanted. Using athymic rats could introduce confounding variables, especially in a traumatic injury model.

3. Figure 9 and Figure 10, NMJ analysis - longitudinal section or whole-mount images are more informative. NMJ could be partially innervated, fragmented, or poly-innervated and cross-sections of muscle do not reveal dynamic changes of NMJ. How many NMJs were quantified? The percentage in Figure 10 represents per area? The quantification method should be explained in more detail.
In addition, at least 20 - 30 junctions per biological sample should be quantified. The term "mature NMJ" is misleading. If presynaptic terminal and postsynaptic AChR overlap, it means that it is innervated. The mature neuromuscular synapse develops into a pretzel-like shape from round closed boutons. Skeletal muscle nAChRs don't express alpha7 subunit. alpha-bungarotoxin binds to 2 alpha subunits in myofibers.

4. Increased satellite cell migration is a central claim of the manuscript but is not directly shown. An increase in Pax7+ cells near the implant could be due to satellite cell proliferation. Quantification of centrally nucleated fibers or embryonic myosin heavy chain staining in the area surrounding the injury would be helpful to put in context the observed increase in satellite cell number.

5. Were there differences in muscle mass?

6. Do the cells seeded in the construct survive? C2C12 myoblasts/myotubes will not fuse with host muscle, does implanted motor neurons sprout and form NMJ with host muscle?

Minor comments.
o Page 4, line 12: It is overstated to say “acellular scaffolds cannot address such multidimensional challenges” with the current citation. It would be more accurate to say they have previously been unable to address them. Incorporating cells adds major translational challenges and limitations.

o Figure 7: Pax7 staining does not always appear co-localized with nuclei? Please change the color from purple. E and e’ don’t look like they match.

o There are a few typographical errors throughout the manuscript.

We thank the editors and reviewers for their constructive feedback which significantly improved the quality of the work. A detailed response to reviewers' specific comments is available in the following sections (in blue font) for their perusal. However, since there were some common concerns raised by the reviewers, we would like to highlight the major modifications we have performed to address those here:

1. As suggested by the reviewers and editors, we have significantly expanded the scope of the study by enrolling an additional cohort of animals to evaluate the effects of our pre-innervated tissue engineered muscle constructs out to 3-weeks following implant for VML repair in rats. This additional enrollment has added extensive qualitative and quantitative data demonstrating the efficacy of our pre-innervation strategy, as detailed below.

2. We also performed longitudinal non-invasive ultrasound monitoring of injured TA muscle repaired with pre-innervated tissue engineered muscle (compared to control groups) over 3 weeks post-implant. This revealed an exciting finding that our novel pre-innervated muscle was the only approach the resulted in an elevated TA volume over these time points. Although ultrasound has been used in clinical studies of VML, to our knowledge this is the first report of using ultrasound in a rodent model of VML.

3. Pre-innervated tissue engineered muscle constructs facilitated graft revascularization and significantly increased satellite cell and neuromuscular junction density around the injury site, as well as maintained muscle volume up to 3 weeks in vivo as compared to non-pre-innervated myocyte constructs and nanofiber scaffolds alone.

We believe that addition of longer-term data significantly strengthens the results and increases the impact of the paper to the field.

Reviewers' comments:

Reviewer #1 (Remarks to the Author):

The authors have submitted a well-written manuscript that assesses the effect of implanted neuromuscular constructs on the acute regenerative response after VML injury. The constructs were composed of an aligned nanofiber scaffold, C2C12 muscle progenitors, and rat spinal cord motor neurons. The constructs were implanted into an athymic rat TA muscle VML model. The authors report an improvement of in vitro parameters of muscle fiber development and formation of NMJs, which are thought to support further observations in vivo of implanted cell survival and improved vascularization, satellite cell presence, and NMJ formation within the remaining tissue in close vicinity to the implant. Unfortunately, the manuscript only includes a subacute time point (7d post injury) and does not demonstrate a physiological effect of the reported observations. Without this determination it is difficult to understand the significance of the proposed findings, which are based solely on histological
analyses that bear inherent sampling limitations. Moreover, the observations are

nuanced, in that the observations of increased satellite cell, vascularization, and NMJ formation are observed in a region spatially distinct from the implanted material, which is a very different from the purpose/mechanism proposed in the introduction. At the same time, the defect area is devoid of these regenerated structures and there is no direct evidence of cell survival, in particular myocyte/fiber presence in the defect area. Please see the specific comments listed below.

Specific Comments:
1. Introduction 3rd Paragraph: please clarify what “random fiber recruitment” is.

Response - Scaffolds which do not provide topographical guidance (like decellularized ECM based scaffolds) have been found to lead to recruitment of myofibers without specific alignment. Such random orientation of the myofibers can hinder regeneration following application of these scaffolds in a VML model. We have now changed the phrase to “randomly oriented fiber recruitment”.

2. Introduction Final Paragraph: When describing the study, there should be better distinction regarding the expected nature of satellite cell, vasculature, NMJ observations. Specifically, the graphical abstract seems to hypothesize that the changes are expected to occur in the area near the defect and not with the implanted material. This is mostly at odds with the bulk of the introduction that speaks to implanting a construct that will directly promote muscle fiber regeneration. Though unstated, the approach seems to focus more on implanting a construct that improves the survival or repair of the muscle tissue that is left after the initial injury.

Response - We thank the reviewer for this valuable insight. It is correct that our study is focused on the implications of pre-innervation on the regenerative milieu of the remaining host muscle. In order to highlight this aspect, we have modified the first and last paragraphs of the Introduction Section.

3. Figure 2/Co-culture: Did you observe spontaneous contraction of the myotubes after differentiation? The terminology using bundles requires definition and clarity. The data do not support the formation of muscle fibers and certainly not bundles of fibers. Within the figure, please consider having panels a and b aligned in the same direction. Are these panels imaged at different magnification- the scale bars are different sizes?

Response - These are excellent points. Yes, we did observe spontaneous contractions of myotubes when they were cocultured with spinal motor neurons (see new Supplementary Videos included with the resubmission). We have also now changed Fig 2 to include myosin heavy chain staining data as well as a volumetric view obtained from multiple Z-stacks to allow better appreciation of neuromuscular bundle formation. Panels a and b are now aligned in the same direction and they have the same Scale bars. Appropriate Scale bars are included for the other panels.

4. Figure 3. The imaging appears over-exposed and does not allow observation of structural detail. Did the authors verify the phalloidin stain with a sarcomeric actin? Why is the density of cells in panel b for MN-MYO greater than that in panel a? For the fusion index, how were individual myotubes discerned to make the analysis? For panel b, please include labels for the staining like in panel a.

Response - As suggested, we have reduced the exposure of the image in an effort to bring out structural details. As noted above, we verified the phalloidin stain with myosin heavy chain (see Fig 2d). The cell density in panel "a" is lower to allow visualization of discrete NMJs which becomes difficult in a very dense culture. For calculating MFI, the margins of individual myofibers stained with Phalloidin were marked along the length of the fibers and the nuclei number was calculated – this is clarified in the Methods. As suggested, we have now included staining labels in panel "b".

5. Figure 5. The sizing of the imaging should be consistent across groups in this figure and throughout the paper. Panel a: there appears little phalloidin staining within the defect raising question regarding the survival of implanted cells. Does the first image have four colors, with a blue channel for nuclei? If so, the lack of nuclei within the defect does no support cell survival. Panel b: The defect has greater phalloidin stain than the co-culture- this is not commented on the results. Additionally, in panels a and be there appears to be fibrous encapsulation that warrants discussion. Panel c: is concerning because it would seem that the muscle nearby should still show positive staining for phalloidin, as in panel b and even panel d. The lack of this positive staining precludes confident analysis of these findings.

Response - We apologize for lack of clarity in Fig 5 of the original submission, which had demonstrated cell survival using cross-sectional images from all the groups. We decided to remove Fig 5 of the previous version and in the present version of the manuscript, we use what we believe to be much clearer demonstrations of cell survival using longitudinal sections as shown in Fig 6 and Supplementary Fig 1. We believe that these new figures clarify and address the individual points raised above. Also, we have ensured that the sizing of images is consistent across groups in the various figures.

6. Figure 6. The laminin staining is perplexing, in that it is unclear why the staining did not also surround muscle fibers in the remaining muscle and only stains the layers of the implanted scaffolds. In the methods, please specify the type of laminin that was used to coat the scaffold. The images are overexposed and do not allow visualization of structure. There are no structures in the images that are obviously muscle fibers.

Response - These are excellent points. Unlike crush injuries, VML leads to loss or injury to the basal lamina in skeletal muscles. Laminin - being one of the major constituents of the basal lamina – may therefore be removed from the damaged basement membrane.

This could be a reason why some (not all) of the myofibers near the injury site appear to have reduced laminin. Moreover, this apparent loss of laminin in the surrounding muscle fibers appeared to be more pronounced at 1 week as compared to 3 weeks post injury; of note, a 1 week time point has rarely been explored in the literature. We now address these important points in the Discussion.

As noted above, we believe that our additional figures better allow visualization of the underlying host muscle structure. For instance, we believe that longitudinal sections in Fig 6 illustrates the presence of long aligned myofibers (MHC+) and axons (NF+) along the alignment of the nanofiber sheets (Laminin+). Also, the cross-sectional images in Supplementary Fig 2b show some host myofibers containing laminin.

Finally, we used Mouse Laminin to coat the nanofiber sheets. Details are included in the Methods section.

7. Figure 7. The analysis area cannot be estimated as it is unclear where the defect is. The panels appear to be taken at different magnification, specifically panel B appears at higher mag than the others? Panel e, the skeletal muscle actin stain is blown out and e' does not appear to show the dapi or hoescht stain for pax7 negative nuclei (?). Again, please provide similar sized images for comparison of like data.

Response - Thank you for bringing these points to our attention. We have modified many of the figures, including Figure 7, to include our findings from a new cohort of animals evaluated at 3-weeks following VML repair. However, the images for each group in Fig 7a only show representative clusters of satellite cells near the injury site and not the full 5mm^2 area sampled for the analysis. To address this point, we have now included a separate figure (new Supplementary Figure 2c) to show the full analysis area and how the quantification ROI was selected with respect to the defect region.

For clarity, we have arranged Fig 7-10 in the following manner – Panel a-b: provide representative images at identical magnification; Panel c: A high magnification image to provide better appreciation of the structure(s) being quantified (representative of the magnification used for counting); and Panel d-onwards: Quantified data as graph(s).

Finally, as requested, we have reduced the exposure of the actin stain and used white arrows to denote $Pax7^-/HST^+$ nuclei. As noted above, all images in the same panel are of identical magnification.

8. Figure 8. The analysis is described as occurring 100 micron from the edge of the defect. Again, the images are of different mags making the quick comparison difficult. The error bars are 100 micron. Visualization of this distance reveals little to no vessels in any of the groups. Greater methodological clarity is need to assess what is quantified and compared.

Response - We believe that our response to the previous point has addressed this concern. We have also provided further detailed methodology about defining microvessels and their quantification in the Methods and in the related Figure caption.

9. Figure 9. Please provide similar size and mag images. From the images, it is not possible to know where the defect is and to ascertain where 100 micron from the defect edge is. Thus, it is not possible to know what is counted for the analysis.

Response - As addressed above, all images within a like panel are now of identical size. As noted above, a new Supplemental Figure details the position of the quantified region relative to the defect edge, along with better definition(s) of the structure(s) counted for analysis.

10. Figure 10. Please provide similar size and mag images. The images do not support the observation of motor endplates with pretzel like morphology.

Response - As addressed above, all images within a like panel are now of identical size. In addition, pretzel shaped morphology of NMJs can be better visualized in the high resolution image provided, hence we would like to refer the reviewer to Fig 10b for an example of the structures identified and quantified across the groups.

11. The methods need to include a detailed description of staining control studies performed and imaging procedures to control for intensity etc.

Response - We thank the reviewer for bringing this to our attention. We have now included these additional details in the Methods in the last paragraph of section on immunohistochemistry.

Reviewer #2 (Remarks to the Author):

Drs. Das and Cullen et al present a new method of combining nerve and muscle cells on a biomimetic scaffold to improve recovery from VML. This innovative new method builds upon prior research that has pre-seeded scaffolds with myocytes, endothelial or stem cells but tries to create more mature innervated scaffolds prior to implantation with combined myocyte and motor neuron transplants. While overall this is an intriguing new method, several concerns remain about the current version of the paper.

1) In Fig. 5, the group evaluates ChAT and NF-200 in vivo but do not stain for this in vitro. It is difficult to interpret the meaning of these stains within the sheets (are they increasing in number or decreasing once implanted) without baseline numbers from in

vitro staining. In addition, quantification of these stain would be necessary to determine significant changes.

Response - This is a valid point, and we thank the reviewer for bringing it to our attention. We generally use Tuj-1 as a pan-axonal stain in vitro to visualize all axons. For tissue samples we generally use more matured markers like Neurofilament and ChAT (matured motor neuron marker) to minimize background. Hence the disparity in antibodies used for in vitro and in vivo parts of the manuscript.

However, to address this specific point, we have included in vitro and in vivo staining examples with muscle specific Myosin Heavy Chain (Fig 2d, Supplementary Fig 2a) and motor neuron specific ChAT (Supplementary Fig 1). A quantitative assessment of these stains is challenging due to the heterogenous distribution of cells within the sheets. This is further complicated by the curling of the nanofiber sheets in vivo and during tissue sectioning making it difficult to accurately identify and discern discrete structures. Hence, we have kept our cell survival analysis purely qualitative. However, we do agree that this is a limitation of the present study and we will keep this mind during future experiments.

2) In Fig 6, it is difficult to determine if the stains point to survival of pre-implant MN-MYO group given the proper negative controls are not shown in the figure or as supplements to determine qualitative differences.

Response - We thank the reviewer for bringing this to our attention. Fig 6 is now supported by Supplementary Fig 2 which shows a comparison of cell survival (MHC and NF staining) between MN-MYO and MYO groups.

3) The paper would be strengthened by analysis at longer duration (typically at least 21 days in the muscle literature). This later time point is especially important given the point mentioned in the discussion about satellite cells and lack of their presence on the scaffold. Since these cells have been present on other scaffolds at these early time point, a later point would demonstrate if eventually these important components of muscular regeneration eventually do migrate onto the scaffold.

Response - We agree with the reviewer and, as requested, have significantly expanded the study to include a cohort survived to 3 week following implantation. We did not observe satellite cell migration within the scaffold even after 3 weeks, but we did notice that pre-innervation facilitated significant graft revascularization. Additionally, pre-innervation was found to enhance muscle volume recovery as well as promote NMJ formation around the injury site.

4) In the discussion, the authors mention that no signs of inflammation based on gross pathology were observed. To consider this point fully, further stains (such as H&E or specific inflammatory antibody staining) to evaluate for routine inflammatory response

should be included. Otherwise, it is difficult to conclude the inflammatory response to the implant and this should be removed from the discussion.

Response - We thank the reviewer for raising this point. Inflammation is a major player in the progression of VML. Detailed study on the implications of pre-innervation on the inflammatory cascades following VML is beyond the scope this manuscript but is an area we are interested in for future work. However, keeping in mind the importance of inflammation, we performed preliminary staining with a pan macrophage marker (CD 68). Although there was limited $CD68^+$ structures after 1 week (data not shown), we did see somewhat uniform amount of macrophage response across all groups after 3 weeks as shown in a new Supplemental Figure 3.

Minor point: A reference for prior validation of MFI should be included in the method section.

Response - We have now included multiple references for fusion index.

Reviewer #3 (Remarks to the Author):

The manuscript by Das et al is an interesting study, in which the authors have created a novel bioengineered construct combining myoblasts and motor neurons for implantation into an established VML defect. This study addresses a current gap in bioengineered VML strategies which often lack focus on re-innervation of the critically injured muscle. However, there are several issues that need to be addressed before being considered for publication.

Major Concerns
1. The goal or the hypothesis of the study is not clearly stated. As all analyses were done on tissue proximal to the implant, rather than within the implant itself, it is unclear whether the purpose of the implant was to directly integrate with the host muscle or to act as a source of positive paracrine signaling.

Response - We thank the reviewer for raising this valid point. Our study is focused on the implications of pre-innervation on the regenerative milieu of the remaining host muscle. To make this point explicitly clear we have now modified the first and last paragraphs of the Introduction section.

2. The majority of the analyses could have been done with congenic rats.
Myoblast can be isolated from rats and embedded in the construct along with motor neurons and then construct can be implanted. Using athymic rats could introduce confounding variables, especially in a traumatic injury model.

Response - We thank the reviewer for the valuable suggestion. However, we respectfully assert that performing the entire study again with congenic animals would be a great follow-on study but is beyond the scope of the current manuscript. We will therefore consider this point while planning future experiments.

3. Figure 9 and Figure 10, NMJ analysis - longitudinal section or whole-mount images are more informative. NMJ could be partially innervated, fragmented, or poly-innervated and cross-sections of muscle do not reveal dynamic changes of NMJ. How many NMJs were quantified? The percentage in Figure 10 represents per area? The quantification method should be explained in more detail.
In addition, at least 20 - 30 junctions per biological sample should be quantified. The term "mature NMJ" is misleading. If presynaptic terminal and postsynaptic AChR overlap, it means that it is innervated. The mature neuromuscular synapse develops into a pretzel-like shape from round closed boutons. Skeletal muscle nAChRs don't express alpha7 subunit. alpha-bungarotoxin binds to 2 alpha subunits in myofibers.

Response - We thank the reviewer for the valuable suggestions. Whole mount imaging for the animals, while extremely informative, is non-trivial and we suggest that establishing this technique is beyond the scope of this present study. We appreciate the suggestion and will consider this for our future experiments.

We do agree that the present study does not go into qualitative details of NMJs. Although some studies have reported such analysis, most of the VML related literature only focus on NMJ counts. We respectfully suggest that our methodology is consistent with the existing state of the art in terms of AchR and NMJ counts.

However, we have included more details about how many AchRs/NMJs were counted under IHC Methods section (last paragraph).

We agree with the reviewer that there is a greater burden to assert "mature" NMJs and have now replaced "mature NMJs" with only "NMJs" throughout the Results and Discussion.

We thank the reviewer for pointing out that Skeletal muscle nAchRs do not express alpha-7 subunit. We have modified the sentence under Results section to indicate Bungarotoxin binds to alpha 1 subunit of nAchRs.

4. Increased satellite cell migration is a central claim of the manuscript but is not directly shown. An increase in Pax7+ cells near the implant could be due to satellite cell proliferation. Quantification of centrally nucleated fibers or embryonic myosin heavy chain staining in the area surrounding the injury would be helpful to put in context the observed increase in satellite cell number.

Response - This is a valid point raised by the reviewer and we agree that our data do not conclusively show migration or proliferation of satellite cells. We have hence modified the

text to indicate more precisely that we observed "increased density of satellite cells" around the injury site after 1 and 3 weeks of VML repair with pre-innervated constructs.

We did not perform quantification of centralized nuclei in this study but will consider it for ongoing and future studies.

5. Were there differences in muscle mass?

Response - To address this concern, we are pleased to include additional data showing in vivo measurements of muscle volume as assessed using ultrasound (see new Fig 5). This revealed an exciting finding that our novel pre-innervated muscle was the only approach the resulted in an elevated TA volume over these time points. We believe that this methodology provides an accurate, longitudinal measure of TA cross-sections, and is also a highly translational technique for VML patients.

6. Do the cells seeded in the construct survive? C2C12 myoblasts/myotubes will not fuse with host muscle, does implanted motor neurons sprout and form NMJ with host muscle?

Response - This is an excellent point. Yes, the implanted C2C12 cells and spinal motor neurons survive up to 3 weeks in vivo (See Fig 6 and Supplementary Fig 2a-b). We did not find any evidence of our implanted myocytes fusing with host myofibers nor the motor neurons sprouting into the host muscle. We have added text to the Results and Discussion to specifically address these points.

Minor comments.
o Page 4, line 12: It is overstated to say "acellular scaffolds cannot address such multidimensional challenges" with the current citation. It would be more accurate to say they have previously been unable to address them. Incorporating cells adds major translational challenges and limitations.

Response - We have modified the sentence as per the reviewer's suggestion.

o Figure 7: Pax7 staining does not always appear co-localized with nuclei? Please change the color from purple. E and e' don't look like they match.

Response - We have modified the Figure to include Pax-7/HST channel showing colabelling of Pax-7 and HST for satellite cells. *We have kept the color of Pax-7 as purple to be consistent with other figures where the structure of interest is purple (satellite cells, AchRs, NF^+ axons and MHC^+ myocytes)*

o There are a few typographical errors throughout the manuscript.

Response - We have checked the manuscript thoroughly and removed typographical errors.

REVIEWERS' COMMENTS:

Reviewer #1 (Remarks to the Author):

The authors have adequately addressed my initial comments. I have no further comments.

Reviewer #2 (Remarks to the Author):

We appreciate the substantial experiments and data that was added to the research study to strengthen the work and its conclusions. They have addressed all of significant concerns with these experiments and clarifications.

Reviewer #3 (Remarks to the Author):

The authors have adequately addressed all of my concerns. I believe the revised version of the manuscript is suitable for publication.
